# Study of the impact of long-duration space missions at the International Space Station on the astronaut microbiome

**DOI:** 10.1038/s41598-019-46303-8

**Published:** 2019-07-09

**Authors:** Alexander A. Voorhies, C. Mark Ott, Satish Mehta, Duane L. Pierson, Brian E. Crucian, Alan Feiveson, Cherie M. Oubre, Manolito Torralba, Kelvin Moncera, Yun Zhang, Eduardo Zurek, Hernan A. Lorenzi

**Affiliations:** 1 Department of Infectious Diseases, J. Craig Venter Institute, Rockville, MD USA; 2 NASA-Johnson Space Center, Houston, TX USA; 3 Universidad del Norte, Barranquilla, Colombia

**Keywords:** Microbiology, Computational biology and bioinformatics

## Abstract

Over the course of a mission to the International Space Station (ISS) crew members are exposed to a number of stressors that can potentially alter the composition of their microbiomes and may have a negative impact on astronauts’ health. Here we investigated the impact of long-term space exploration on the microbiome of nine astronauts that spent six to twelve months in the ISS. We present evidence showing that the microbial communities of the gastrointestinal tract, skin, nose and tongue change during the space mission. The composition of the intestinal microbiota became more similar across astronauts in space, mostly due to a drop in the abundance of a few bacterial taxa, some of which were also correlated with changes in the cytokine profile of crewmembers. Alterations in the skin microbiome that might contribute to the high frequency of skin rashes/hypersensitivity episodes experienced by astronauts in space were also observed. The results from this study demonstrate that the composition of the astronauts’ microbiome is altered during space travel. The impact of those changes on crew health warrants further investigation before humans embark on long-duration voyages into outer space.

## Introduction

Astronauts spending six months to a year at the International Space Station (ISS) undergo a wide variety of stresses that can impact crew health and productivity. These stresses range from environmental factors (e.g. microgravity and increased radiation) to social stresses (e.g. isolation, anxiety and sleep deprivation)^1–4^. Space flight presents the further danger of isolation from medical experts, making preventative measures to ensure astronaut health paramount. For decades NASA has studied the effects of space travel on humans and has sought to identify factors that can be regulated to improve the chances of astronauts returning to Earth healthy^3–7^. As the duration of space missions increases, it becomes more and more important to maintain long term health in space to keep astronauts functioning at a high level^3,8,9^.

Previous inflight studies have shown that astronauts have reported a range of health issues ranging from GI distress, respiratory illness and skin irritation and infections^10,11^. Many of these symptoms have been associated with a weakening of the immune function as shown by reactivation of Epstein Bar Virus (EBV), and Varicella Zoster Virus (VZV) during space flight^12,13^. Altered production of cytokines including increases in white blood cell counts have been measured during space flight and associated with altered adaptive immunity^14^. Further studies have shown astronauts experience immune dysregulation, changes in neutrophil functions, and neuroimmune response during space flight^15–17^. A recent study documented a persistent skin rash in an astronaut at ISS that also correlated with immune dysregulation^18^. In addition to physiological changes and reductions in immune response experienced by astronauts, microgravity can also cause changes in homeostasis and microbial biochemistry^19–21^.

Terrestrial studies of the human microbiome suggest that many of the maladies experienced by astronauts can be caused or exacerbated by microbiome dysfunction. Early astronaut microbiome studies documented the diversity of microbes associated with astronauts and the transfer of the pathogen *Staphylococcus aureus* from one astronaut to another^22,23^. While these early studies collected a wide range of samples, they were limited to investigating the small fraction of organisms that could be grown in pure culture. This study documented the diversity of microbes associated with astronauts before, during and after long duration space missions at the ISS using culture independent 16S rRNA gene analysis. The evidence presented herein shows that space travel can have both transient and longer lasting impacts on the microbiome of astronauts, and that these changes are associated with alteration of immune function.

## Results

### Sample collection and processing

**Figure 1:**
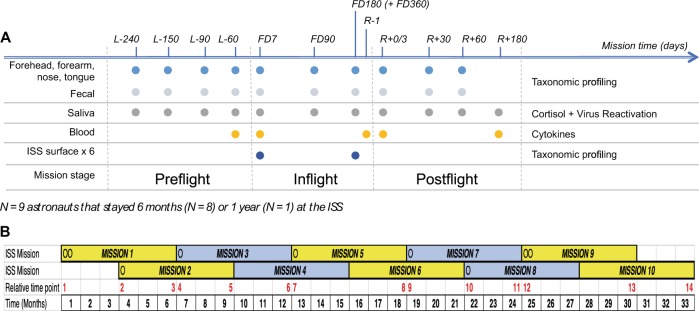
Study experimental design. (**A**) Schematic representation of study experimental design. Sample collection time points are indicated on top of the scheme. Number prefixes indicate days before launch (L−), during flight in the ISS (FD) and before (R−) and after (R+) the return to Earth. Colored circles depict time points used for collection of samples specified on the left of the figure. Data types generated from samples are shown on the right. (**B**) Diagram depicting space missions scheduled during the study. Relative mission time points, in red, indicate the order in which inflight time points FD7 and FD180/FD360 were collected along the study. Circles denote mission start time for each astronaut that participated in this study. Yellow and grey boxes represent the six-month periods astronauts stayed in the ISS. Black numbers indicate the time in months a mission at the ISS started and ended since the beginning of the first ISS mission that contributed to the study.

To determine the effects of spaceflight on the human microbiome, we performed a longitudinal study on nine astronauts spending from six to twelve months at the ISS. During the study, astronauts collected microbiome samples from their forehead and forearm skin, their two nostrils, tongue and stool, as a proxy of the gastrointestinal tract (GI) microbiota, at ten different time points before, during and after the mission (see Fig. 1). In addition, the crew collected in-flight microbial environmental samples from six different surfaces and one sample from the water reservoir in the ISS to study the interplay between the ISS microbial communities and the crew’s microbiome. Thereafter, we extracted the DNA from all microbial samples and sequenced the V4 region of the bacterial 16S rRNA gene to assess their microbial composition. Also, to investigate possible interactions among astronauts’ stress levels, immune response and their microbiota, each astronaut collected saliva to measure cortisol levels and virus reactivation as well as blood samples to evaluate cytokine concentrations in plasma (see Fig. 1).

**Figure 2:**
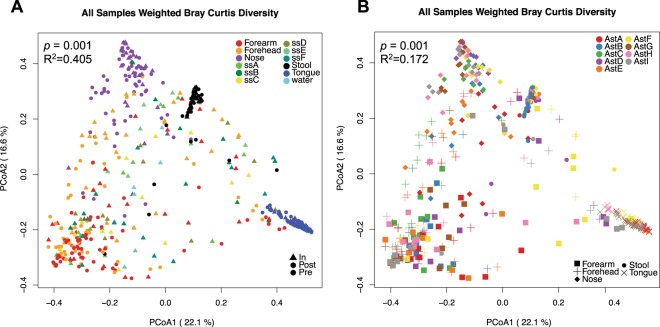
Principal coordinate plots of weighted Bray-Curtis beta diversity distances across all samples collected in the study. (**A**) Samples colored by body or ISS surface site; The permanova p-value and R^2^ using body site as the independent variable is shown. (**B**) Human microbiome samples colored by astronaut identifier. Each microbiome is represented with a different symbol. Permanova p-value and R2 using astronaut identifier as the independent variable and stratifying by body site are indicated.

Characterization of the overall spatial patterns of microbial community composition by Principal Coordinate Analysis (PCoA) of beta diversity dissimilarity values showed that the factor that most contributed to variation among samples was sampling site (p = 0.001, Fig. 2A and Supplementary Fig. S1). These results agreed with previous studies showing that the skin and nose microbiomes are more similar to each other than to those from feces or mouth^24,25^. In addition, samples collected from the same body site tended to cluster by crew member (p < 0.001 for each of the five body sites, Fig. 2B and Supplementary Figs S1 and S2). Interestingly, ISS environmental samples overlapped with specimens from the two skin sites and the nose microbiota. Further analysis showed that there was no significant difference among bacterial species that were present/absent in ISS microbial communities and inflight forehead and forearm skin microbiomes (unweighted beta diversity, Supplementary Fig. S3).

### Impact of space travel on the alpha diversity of the astronaut’s microbiome

**Figure 3:**
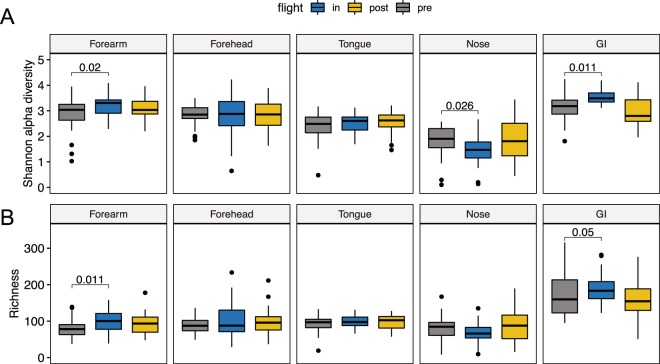
Changes in alpha diversity and richness of the astronauts’ microbiome. Boxplots depicting changes in Shannon alpha diversity (**A**) and Richness (**B**) of the five human microbiomes surveyed in this study during a mission to the ISS. Significant linear mixed-effects model p-values are depicted using mission stage Preflight as baseline.

To measure changes to the diversity of crew microbiomes during space-travel we performed comparative analysis of the Shannon alpha diversity index and richness for each of the five human body locations surveyed (Fig. 3). This analysis showed no alteration in alpha diversity or richness of the tongue microbiota associated with space travel. However, in the GI, Shannon alpha diversity and richness significantly increased in space and returned to their baseline preflight levels after crew members return to Earth (Fig. 3). The only exception was AstB, whose GI microbiota had the highest preflight and postflight alpha diversity and species richness levels among all astronauts and did not significantly change over the course of the mission (Supplementary Fig. S4). In addition, we observed a significant reduction in alpha diversity of the nares microbiome during space flight that returned to preflight levels after returning to Earth. Contrary to alpha diversity, overall species richness of nasal microbiota did not change significantly during the mission, indicating that the inflight changes in alpha diversity were due to a reorganization of the relative abundance of microbial taxa (Fig. 3).

Initial analysis of the two skin sites surveyed, forearm and forehead, revealed no consistent trend in the way the ISS environment affects the skin microbiota of individual crew members (Figs 3 and S4). While Shannon alpha diversity and richness became significantly higher in the forearm skin during spaceflight, no change was evident in forehead skin samples. However, further inspection at the subject level showed that in five out of the nine astronauts’ forehead and forearm samples, skin alpha diversity and richness became significantly higher in space (Supplementary Figs S4 and S5) and remained elevated at least 60 days after their return to Earth. In the remaining four astronauts, these two indexes showed a downward trend in space that changed to preflight levels after astronauts returned from space, except for forehead alpha diversity (Supplementary Figs S4 and S5). Notably, within the same subject, forehead and forearm skin microbiome samples showed similar upward or downward shifts (Supplementary Fig. S4).

### Changes in beta diversity of the astronauts’ microbiota

**Figure 4:**
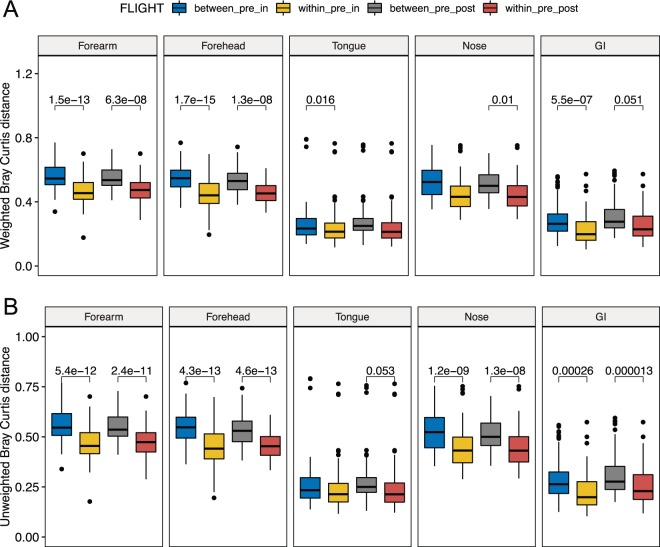
Changes in beta diversity of the astronauts’ microbiome. Boxplots showing differences in weighted (**A**) and unweighted (**B**) Bray-Curtis beta diversity distances among microbiome samples within and between mission stages. Within_pre_in, within preflight and within inflight distances; between_pre_in, distances between preflight and inflight samples; between_pre_post, distances between preflight and postflight samples; within_pre_post, distances within preflight and within inflight samples. Linear mixed-effects model p-values ≦ 0.06 are depicted using within_pre_in or within_pre_post as baselines.

To measure overall changes in astronaut microbiome composition associated with space travel we performed comparative weighted and unweighted beta diversity analyses of taxonomic profiles between pre, in and postflight samples (Fig. 4). This comparison revealed inflight qualitative (unweighted) and quantitative (weighted) changes in the microbial composition of the GI and skin microbiomes that persisted in postflight samples (Fig. 4). Spaceflight-associated changes in the nares microbiota were only significant when considering qualitative differences in the microbial composition between pre and inflight samples. These changes remained significant for at least 60 days after the return to Earth. The tongue microbiota presented inflight changes associated with shifts in the relative abundance of bacterial species, but these quantitative differences disappeared once the crew returned to Earth (Fig. 4).

To determine the influence that the amount of time spent at ISS has in any observed changes in astronaut microbiomes, we compared inflight and postflight time points to all preflight time points (as a baseline) using the mean Bray-Curtis (BC) dissimilarity distance (Supplementary Fig. S6). This analysis showed that compositional changes of the nose, skin and GI microbiomes were rapid and became evident by FD7. These changes persisted for at least six months until the end of the mission at the ISS. Furthermore, beta diversity changes did not significantly increase with the time astronauts spent in space, although preflight-inflight BC distances of the two skin sites and the nose microbiota showed a very modest upward trend associated with time spent inflight (Supplementary Fig. S6). In addition, compositional shifts in the skin and nose microbiomes persisted for at least 60 days after the astronauts returned to Earth. However, the composition of the GI microbiota became similar to preflight samples within two months of the astronauts’ return from the ISS.

Interestingly, PCoA plots of weighted dissimilarity distances showed that the GI microbiome became more similar across astronauts in space compared to preflight samples (Supplementary Fig. S7). The only exception was AstB, whose GI microbiome also had the highest preflight alpha diversity and richness values (Supplementary Fig. S4). This inflight similarity tended to dissipate once astronauts returned to Earth but remained significantly closer than preflight samples within two months of the return to Earth.

### Alteration of the microbial composition of the crew microbiome associated with the space environment

**Figure 5:**
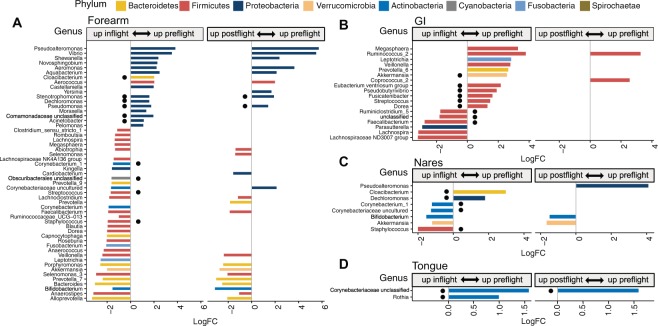
Changes in the relative abundance of bacterial genera of the astronauts’ microbiome. Changes in the microbiota of the forearm skin (**A**), GI (**B**) and nose (**C**) during a mission at the ISS and after the return to Earth. Colors represent different phyla; horizontal axis, logarithm of the fold change in relative abundance between preflight and inflight or postflight samples; vertical axis, bacterial genera. Black circles indicate genera belonging to the corresponding preflight core microbiome.

To investigate the influence of the ISS as a contained and human built environment on the crew microbiomes, differential abundance analysis of bacterial taxa was performed between samples collected before, during and after the mission to the ISS (Fig. 5, Supplementary Fig. S8 and Table S5). In addition, for each of the five crew microbiomes, we defined a core microbiome, composed of all bacterial taxa that were represented in at least 75% of all preflight samples (Supplementary Table S6). This analysis identified 17 gastrointestinal genera whose abundance significantly changed in space (adjusted p-value by the false discovery rate (FDR) < 0.05). Thirteen out of the 17 genera belonged to the Phylum Firmicutes, mostly to the order Clostridiales, with nine genera being part of the GI core microbiota (Fig. 5B and Supplementary Table S5). Among these taxonomic groups, there was a more than five-fold inflight reduction in *Akkermansia and Ruminococcus*, and a ~3-fold drop in *Pseudobutyrivibrio* and *Fusicatenibacter*. Most of these compositional changes reverted to preflight levels after astronauts returned to Earth, with the exemption of two genera of the phylum Firmicutes.

Examination of skin samples also revealed changes in the relative abundance of several bacterial phylotypes during the space mission, corresponding to 49 and 43 genera in the forearm and forehead respectively, ten of which belonged to the respective core microbiomes (Fig. 5, Supplementary Fig. S8 and Table S5). Noteworthy, skin microbial communities whose abundance decreased in space were mostly Gram-negative Proteobacteria with a predominance of Gamma and Betaproteobacteria. These groups included bacteria from the genus *Moraxella, Pseudomonas* and *Acinetobacter*. In contrast, most of the skin bacteria that became more abundant inflight belonged to the phylum Firmicutes, Bacteroidetes and Actinobacteria, including bacteria of the genus *Streptococcus*, *Staphylococcus* and *Corynebacterium*. Postflight samples showed a similar trend as inflight samples, with lower Proteobacteria and higher Firmicutes, Bacteroidetes and Actinobacteria compared to Preflight skin (Fig. 5 and Supplementary Fig. S8).

In addition, we observed a similar but milder response of the nares microbiota to the space environment with a significant drop in two genera of Gram-negative bacteria that were also reduced in skin (Fig. 5, Supplementary Fig. S8 and Table S5). Likewise, nose inflight samples showed increases in five bacterial genera, four of which became more abundant on the skin of astronauts inflight. Five out of the seven genera that changed during spaceflight were also part of the nares core microbiome. Many of the observed changes in the nares microbiota dissipated after the astronauts returned to Earth, with higher abundance of *Bifidobacterium* and *Akkermansia*, and lower levels of *Pseudoalteromonas* in postflight samples. In the tongue, only two Gram-positive genera, that also belonged to the core microbiome, were found significantly reduced during spaceflight, *Rothia* and an unclassified genus of the family *Corynebacteriaceae*, which also remained reduced in postflight samples (Fig. 5 and Supplementary Table S5).

The analyses of alpha and beta diversity above showed that the GI microbiota of AstB was compositionally different and responded differently to the ISS environment compared to the GI microbiome of the other four astronauts that also collected inflight stool samples. The composition of the GI microbiome of AstB was compared to that of Astronauts C, D, F, and G across either preflight or inflight samples (Supplementary Table S7). Those comparisons exposed three genera that were differentially abundant in both pre and inflight samples between AstB and the other four crew members. Before flight, 23 genera differed between the two groups of samples. One third of them belonged to the families *Lachnospiraceae* and *Ruminococcaceae* and were particularly enriched in the GI microbiome of AstB, while genera from the families *Prevotellaceae* and *Veillonellaceae* were more predominant in the GI of the other four astronauts. Among inflight samples there were 11 distinct genera between the GI microbiota of AstB and the other four crew members, with the GI microbiome of AstB having higher relative abundance of *Akkermansia* and lower levels of *Bifidobacterium* and *Pseudobutyrivibrio*. Contrary to AstB, the GI microbiota of astronauts C, D, F and G became more similar in space. To identify the bacterial species that made the largest contribution to the compositional similarity across astronauts in space, we compared the coefficient of variation (CV) of their relative abundance among preflight versus inflight GI microbial samples. This analysis revealed that only a few bacterial genera, including *Pseudbutyrivibrio*, *Dorea*, *Ruminococcus 2*, *Bifidobacterium*, *Blautia*, *Fusicatenibacter* and *Akkermansia* accounted for more than 90% of the total CV reduction observed in the GI microbiome of AstC, D, F and G during their trip to the ISS (Supplementary Fig. S9).

### Identification of microbial changes associated with Astronauts’ immune dysregulation in space

**Figure 6:**
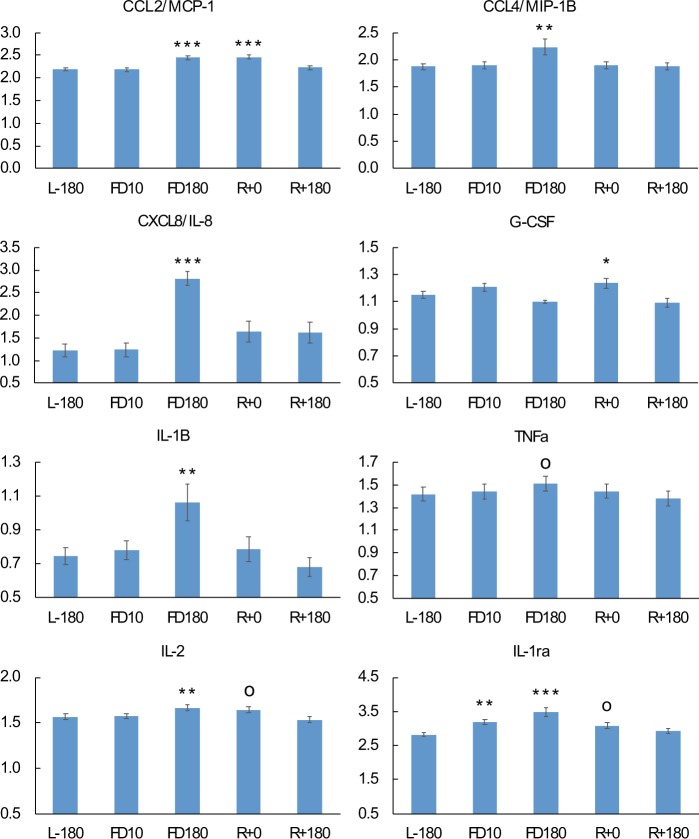
Variation of plasma cytokine concentrations during spaceflight and after the return to Earth. Significance values are with respect to preflight L-180 time point. ^o^p-value < 0.1; *p-value < 0.05; **p-value < 0.01; ***p-value < 0.001.

To gain insights into the potential impact of changes to the microbiome during space flight on immune functioning, changes to cytokine abundance in plasma were compared to changes in the composition of the GI microbiome (Fig. 6, Tables 1 and S3). With control of the false-discovery rate (FDR) to 5%, we found 10 significant changes (p < 0.0068) in cytokine concentrations at various time points relative to pre-flight. By FD10, analysis of plasma cytokine profiles did not reveal any significant change in cytokine concentrations relative to preflight levels except for anti-inflammatory protein IL-1ra that was slightly higher (Fig. 6). However, by FD180 we observed a moderate but significant increase in the concentration of several pro-inflammatory cytokines, MCP-1, IL-8, IL-1b and MIP-1β, and a near significant rise in TNFa (p = 0.0075, FDR q-value = 0.055). In addition, we detected elevated inflight levels of cytokines IL-2 and IL-1ra. Cytokine concentrations reverted to preflight levels within two months of returning to Earth.

**Table 1:** Association between changes in cytokine concentrations and the relative abundance of GI microbiota during a mission at the ISS and the posterior return to Earth.

Cytokine	Cytokine vs GI microbiota correlation (D)	SE	Correlation p-value	Inflight cytokine change	OTU	Genus	Inflight OTU change
CXCL8/IL-8	−0.581	0.144	5.43E-05	↑	Otu000010	*Fusicatenibacter*	↓
IL-1B	−0.622	0.126	8.57E-07	↑	Otu000010	*Fusicatenibacter*	↓
	−0.36	0.087	3.69E-05		Otu000011	*Dorea*	↓
TNFa	−0.467	0.099	2.32E-06	↑	Otu000010	*Fusicatenibacter*	↓
IL-17	0.464	0.114	4.35E-05	ns	Otu000054	*Faecalibacterium*	↑↑↑
IL-1ra	−0.5	0.12	3.09E-05	↑	Otu000011	*Dorea*	↓
	−0.5	0.102	8.89E-07		Otu000028	*Ruminococcus_2*	↓↓↓
IFNg	0.618	0.12	2.85E-07	ns	Otu000038	*Akkermansia*	↓↓
	−0.611	0.06	4.33E-24	ns	Otu000165	*Lachnospiraceae (uncl.)*	↑
IL-2				↑			
IL-4	−0.622	0.155	5.87E-05	ns	Otu000010	*Fusicatenibacter*	↓
IL-10	−0.379	0.103	2.46E-04	ns	Otu001908	*Roseburia*	↓↓
G-CSF	−0.364	0.08	5.67E-06	ns	Otu000016	*Blautia*	↑↑
FGF basic	0.357	0.098	2.85E-04	ns	Otu000054	*Faecalibacterium*	↑↑↑
Tpo	0.352	0.099	3.71E-04	ns	Otu000071	*Lachnospiraceae (uncl.)*	↓↓
VEGF	−0.556	0.087	1.72E-10	ns	Otu000010	*Fusicatenibacter*	↓
	−0.42	0.106	7.08E-05		Otu000011	*Dorea*	↓
CCL2/MCP-1				↑			
CCL4/MIP-1B	−0.38	0.1	1.49E-04	↑	Otu000011	*Dorea*	↓
CCL5/RANTES	0.521	0.138	1.55E-04	ns	Otu000060	*Lachnoclostridium*	↑

D, Somers’ D association coefficient. D > 0 and D < 0 indicate respectively positive and negative associations between cytokine concentration and OTU relative abundance. ns, non-significant change in cytokine concentration during space flight; upward and downward arrows indicate an increase or decrease in cytokine concentration/OTU abundance at the ISS respectively. Number of arrows is proportional to the log2(OTU relative abundance fold-change). Uncl., unclassified genus.

Further correlation analysis identified strong evidence of an association between changes in astronauts’ GI microbiome and changes in cytokine profiles (Table 1). Most notably, the abundance of OTU000010 of the genus *Fusicatenibacter* was negatively correlated with the concentration of pro-inflammatory cytokines IL-8, IL-1b, IL4, and TNFa. In addition, changes in OTU000011 of the genus *Dorea* were also negatively correlated with changes in the level of several cytokines including IL-1b, IL-1ra VEGF, and MIP-1b, all of which were increased in space

### ISS environment and its interaction with the Astronauts Microbiome

The ISS is a relatively closed environment that incorporates microbial communities from two main sources, cargo shipments with very low microbial loads and the astronauts’ microbiota. To investigate the composition of the microbial communities that inhabit the ISS and their interaction with the astronauts’ microbiome, we assessed alpha and beta diversity measurements of the microbiota of six different ISS surfaces (named ssA to ssF, see methods for details) and how they compare to the microbiomes of the crew.

**Figure 7:**
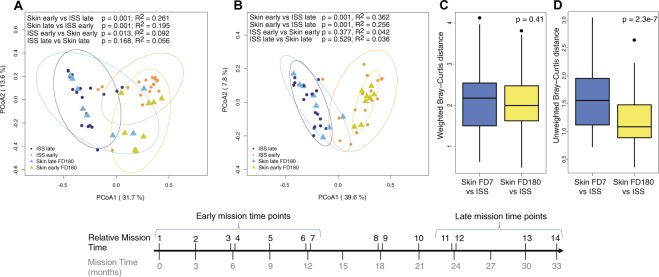
Comparative beta diversity analysis between the astronauts’ skin microbiota and environmental bacteria at the ISS. (**A**,**B**) Principal coordinate analyses of weighted (**A**) and unweighted (**B**) Bray-Curtis beta diversity of FD180/FD360 forehead and forearm skin samples (triangles) and ISS samples (circles) collected during early and late relative mission time points. Ellipses represent 95% confidence intervals. Permanova p-values and R^2^ values are shown. (**C**,**D**) comparative analysis of the difference in mean weighted (**C**) and unweighted (**D**) Bray-Curtis beta diversity distances between skin and ISS samples collected at FD7 (**C**) or FD180 (**D**). Welch two samples t-test p-values for each comparison are shown.

An initial comparative analysis of weighted and unweighted beta diversity distances showed that the microbial profiles of the six ISS sites surveyed were very similar (Supplementary Fig. S10). However, PCoA plots showed that the composition of the six ISS microbiomes significantly changed over time, during the 33 months we surveyed the station, with samples collected at early and late time points forming discrete groups (p = 0.001, Supplementary Fig. S10). Given that the ISS microbiota resembled that of the astronauts’ skin (see above), we hypothesized temporal changes could be influenced by the arrival of new crew members to the ISS every three months. To test this hypothesis, we compared the microbial composition of the ISS samples from early and late time points to the skin microbiome of astronauts that traveled to the ISS at the beginning or end of the study (Fig. 7). This analysis showed that while there were no differences in the species present/absent between skin and ISS samples at either early (p = 0.36) or late (p = 0.5) time points, early skin microbiome samples had a significantly different composition to late ISS microbiome samples (p = 0.001) and vice versa (p = 0.001). Moreover, comparative analysis showed that the mean BC dissimilarity distance between the skin and ISS samples collected by the time astronauts were leaving the ISS (time point FD180) were significantly smaller than the distance between ISS and skin samples collected at FD7, when crew members had just arrived at the ISS (Fig. 7C,D). The similarity between the skin and environmental microbiomes was only evident when considering the type of microbial species present in the skin and the ISS environment (unweighted BC distance), but not their relative abundance (Fig. 7C,D).

**Figure 8:**
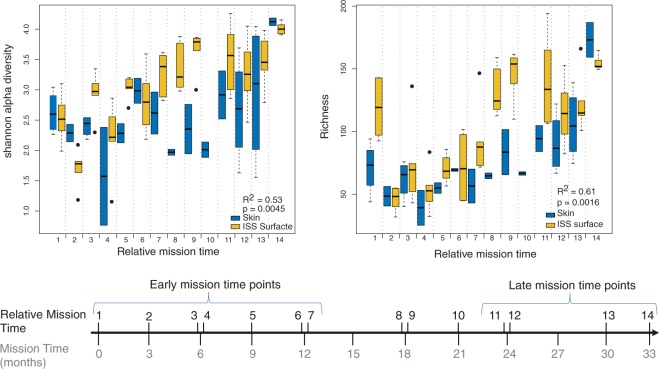
Changes in alpha diversity and richness of astronauts’ skin microbiota and environmental bacteria at the ISS during the entire duration of the study. Changes in Shannon alpha diversity (**A**) and Richness (**B**) of the ISS microbiota over time and their association with Shannon alpha diversity and Richness of forehead and forearm skin samples collected at FD7, FD180 and FD360. Pearson correlation coefficients between skin and ISS samples and correlation p-values are shown.

Comparing differences in microbial alpha diversity and richness revealed that the six ISS sites had similar richness and Shannon diversity values (Supplementary Fig. S11). Alpha diversity and richness were also similar across environmental and inflight skin samples (Supplementary Fig. S11). However, we found that alpha diversity and richness of ISS samples significantly fluctuated over time (Fig. 8) in a manner that correlated with changes in inflight alpha diversity and richness of the crew skin microbiota (Pearson R^2^_Shannon_ = 0.53; R^2^_richness_ = 0.61; Fig. 8A,B).

In spite of the fluctuations in the composition of the ISS microbial communities over time, each of the assessed ISS surfaces had a small proportion of OTUs (<4%) that were highly prevalent during the entire duration of our study, most of them from the phyla Firmicutes, Actinobacteria and Proteobacteria, and therefore, may be long-term residents of the ISS environment (Supplementary Figs S12–S17). In particular, four of these OTUs belonged to the genera *Streptococcus*, *Staphylococcus*, *Corynebacterium-1* and *Cloacibacterium* and were ubiquitously present across all six ISS surfaces and inflight time points.

### Astronauts’ virus reactivation and hormone stress levels

Reactivation and shedding of latent varicella zoster virus (VZV), Epstein bar virus (EBV) and Herpes simplex virus (HSV1) as well as diurnal salivary cortisol, alpha-amylase and dehydroepiandrosterone (DHEA) were measured prospectively in 10 astronauts before, during, and after their mission at the ISS to assess astronauts’ stress (Supplementary Fig. S18 and Table S4)^12^. Two astronauts did not shed any virus in any of their samples collected during the study. VZV reactivation was detected in the saliva of four crew members. Except for AstD, that showed VZV reactivation by L-60, no VZV was detected in saliva before flight. However, VZV was significantly reactivated in space, reaching a peak by FD90. After 30 days of the return to Earth, three of the astronauts became negative for VZV except for AstD that remained positive for up to 180 days (Supplementary Table S4). EBV and HSV1 were detected in the saliva of three and five astronauts respectively, but no association was found between detection in saliva and space flight.

No significant changes in levels of salivary cortisol were detected during the entire mission in any of the crewmembers. The inflight salivary concentration of alpha-amylase, however, was higher than preflight values reaching a maximum by the end of the mission in the ISS. DHEA, showed an opposite trend, becoming less abundant by FD90 at the ISS.

## Discussion

During a space mission, astronauts are exposed to a series of stressors that are likely to affect the composition of their microbiome. Previous studies performed under real or simulated microgravity conditions on culturable commensal and opportunistic pathogenic bacteria suggested that space travel may cause functional changes of the crew microbiome, including bacterial virulence, antibiotic resistance, biofilm formation and growth^26–29^. In addition, studies using culture-based methodologies on astronaut samples collected before and after space flights have identified shifts in the microbial composition of the oral, nasal and intestinal microbiota^30–33^. However, it is likely that these compositional changes are only reflective of the small number of culturable species and were not reflective of overall changes to astronaut microbiomes. To the best of our knowledge, ours is the first project to study the impact of long-term space travel on the astronauts’ GI, skin, tongue and nose microbiomes using 16S rRNA molecular markers from human samples collected in space. Our main goal was to determine the extent of microbiome perturbations experienced during long duration space missions by both astronauts and the ISS environment, and correlate those changes to observed changes in astronaut health. It should be noted though that the nature of this study is exploratory and based on a very low number of participants (n = 9, for stool n = 5) due to the limited availability of astronauts that travel to the ISS. Only six astronauts live and work on the ISS at any given time, and there are huge demands on their time that limit the sampling that can be performed. The low number of participants dramatically reduces the statistical power to detect small fluctuations in astronaut microbiomes and extrapolation of these results to crew members of future space missions should be done with caution. The authors hope that this correlative study will help create a baseline of microbiome changes that can inform future studies of astronauts that include increased sampling of a more diverse range of participants that will allow researchers to draw more robust conclusions and lead to the development of predictive models.

It has been previously proposed that the diversity of commensal bacteria that inhabit different ecological niches of the human body are likely to decrease during long space missions, increasing the risk of opportunistic infections^34–36^. However, our results showed that the Shannon alpha diversity and richness of the GI microbiota significantly increase during six-month missions to the ISS. Also, analysis of stool sample beta diversity showed that compositional differences between pre and inflight GI microbial communities were explained by both shifts in their relative abundance and the acquisition or loss of species during the trip to space (Fig. 4).

Investigations of individual astronauts showed that only four out of the five astronauts that collected stool samples in space exhibited increases in GI alpha diversity while at the ISS. AstB’s GI microbiome remained relatively unchanged over the course of the study and had the highest number of bacterial species among all five astronauts tested (p < 0.05). It is possible that the highly diverse intestinal flora of AstB made it more resilient to perturbations when exposed to the space environment, as has been previously proposed^37^. Our results are also in agreement with a 16S rRNA gene analysis of the intestinal microbiota from mice flown for 13 days on the Space Shuttle Atlantis that did not find any changes in bacterial alpha diversity between pre and postflight stool samples^38^. These results do however highlight that comparative analysis between pre and postflight samples does not reflect the changes to the human microbiome that were observed during space flight. Increasing the frequency and number of participants for inflight sampling should be a priority of future studies.

Analysis of astronaut GI microbiome beta diversity also revealed that the composition of the intestinal microbiota changed in space and tended to become more similar across astronauts, even though not all crew members spent mission time together in the ISS (see diagram from Fig. 1B). It is likely that a driver of this compositional convergence in the GI was the astronauts shifting to the ISS food system, which while diverse (200+ choices), is more similar than the astronauts eating habits on Earth. While many studies have documented rapid microbiome adjustment to changes in diet, the contribution of confounding factors associated with space travel (e.g. microgravity, stress or immune dysregulation) cannot be ruled out.

It is not clear whether the observed alterations in the GI microbiome during spaceflight pose a risk to astronauts’ health. Changes such as the increase in alpha diversity and the relative abundance of *Faecalibacterium* species, a commensal intestinal bacterium associated with a healthy GI tract^39–41^, appear to indicate that the ISS environment has a neutral or beneficial effect on the intestinal health of the crew. However, changes in the relative abundance of *Fusicatenibacter*, *Pseudobutyvibrio*, *Akkermansia* and *Parasutterella*, and the increase of some pro-inflammatory cytokines present conflicting indicators that suggest that increases in alpha diversity and richness during spaceflight are not reflective of an increase in GI health^42,43^. Indeed, our study identified space-associated increases in the relative abundance of *Parasutterella*, which has been positively associated with chronic intestinal inflammation in patients with inflammatory bowel disease (IBD)^44^. In addition, we found a space-associated reduction in the relative abundance of intestinal *Fusicatenibacter, Pseudobutyvibrio* and *Akkermansia*, three bacterial genera with anti-inflammatory properties^45–55^. *Fusicatenibacter saccharivorans* is reduced in the GI of patients with active ulcerative colitis and ameliorates colitis in a mouse model of IBD^45^. Also, our study found that the levels of intestinal *Fusicatenibacter* negatively correlated with the concentration of pro-inflammatory cytokines IL-8 and IL-1b, that were slightly but significantly increased in space (Table 1). *Pseudobutyrivibrio* and *Akkermansia*, two bacterial taxa whose abundance has been found inversely correlated with several inflammatory diseases^48,51–55^, stimulate the production of SCFA in the intestine, which contributes to the integrity of the gut epithelium and reduces the intestinal inflammatory response^46,56^. These results suggest that the observed alterations in the composition of the GI microbiome may contribute to the moderate increase in the inflammatory immune response observed in the crew during space flight. If that is the case, then treatments known to block inflammation in the GI, such as the administration of prebiotics or treatment with the probiotic bacteria *A. muciniphila*, could be implemented in space to reduce the risk of diseases associated with chronic inflammatory responses^57^.

The response of the forehead and forearm skin microbiota to the ISS environment varied among astronauts, with alpha diversity and richness going up or down, depending upon the individual, but was consistent within astronauts between forehead and forearm skin. It is not clear what factors contributed to this differential response to space travel. It is possible that the composition of the microbial communities of the skin, skin-specific properties such as moisture and pH and/or astronauts’ personal hygiene habits have played a role in the differential response of the skin microbiota to spaceflight.

In spite of the bimodal variance amongst astronaut skin microbiomes, we found shifts in the microbial composition that were common across all crew members during space flight. These changes involved a significant inflight reduction of Proteobacteria, mostly Gamma and Betaproteobacteria, with a concomitant increase in Firmicutes, including Staphylococcal and Streptococcal species. It is difficult to predict whether these compositional changes may have deleterious consequences to astronauts’ health. Previous studies have found that patients with atopy have lower Proteobacteria diversity on their skin^58^. In particular, Gammaproteobacteria have been found inversely associated with inflammation and allergy sensitization. Moreover, studies have shown that skin Gammaproteobacteria of the genus *Acinetobacter*, which is reduced in space (Fig. 5 and Supplementary Table S5), positively correlated with the baseline expression of anti-inflammatory IL-10 by peripheral blood mononuclear cells in healthy young individuals^59^ and conferred protection against allergic responses in mice^60^.

Among the most common clinical episodes astronauts experience during space travel are a high frequency of skin hypersensitivity reactions/rashes and skin infections^10^. Therefore, it is possible that the reduction of skin Gammaproteobacteria may contribute to the occurrence of these clinical episodes. It is not clear why Gamma and Betaproteobacteria abundance decreased in the skin of crew members during spaceflight. A study by Ruokolainen *et al*. demonstrated that there is a positive association between the proportion of forest and agricultural lands that compose the environment and the relative abundance of skin Proteobacteria in healthy individuals^58^. Based on that study, it is possible that the lack of a “green” environment plus the constant filtration of environmental air in the ISS contributes to the overall reduction of skin Proteobacteria. Another factor that could contribute to changes in the microbial communities of the skin is the alteration of the skin structure during spaceflight. A pilot study based on a single crew member spending six months in the ISS found a delayed cellular proliferation of the basal skin layer together with a thinning of the upper layer of the epidermis^61^, which could increase the exposure of the microbial communities that reside in the deeper layers of the skin^62^. This change in skin structure might also facilitate the establishment of skin infections by opportunistic pathogens such as Staphylococcal and Streptococcal species that increased during spaceflight^63–65^. While changes in the skin microbiome likely contribute to the high frequency of skin infection episodes recorded in space, the observed correlations cannot inform causation due to a wide range of confounding factors.

Compared to the skin microbiome, fewer inflight changes were found in the nose microbiome. These changes partly resembled those undergone by the microbiome of the skin and were characterized by a drop in both alpha diversity and two Gram-negative bacterial genera together with an increase in five genera including *Staphylococcus, Corynebacterium-1* and *Bifidobacterium*. The observation that no significant change in species richness in the nares microbiome suggests that the lower inflight alpha diversity was due to a less even relative abundance of bacterial species in the nares, rather than to a loss of species. During space travel, astronauts have reported experiencing prolonged congestion symptoms, rhinitis and sneezing^10^. A number of studies have found a higher abundance of Staphylococcal bacteria in the upper respiratory tract of patients with chronic rhinosinusitis^66,67^ and a reduction in diversity^68^. *Staphylococcus* has also been found positively associated with other respiratory diseases, such as allergic rhinitis and asthma^69^. While other factors could be playing a role in the upper respiratory symptoms experienced by the crew at the ISS, an elevated relative abundance of nasal Staphylococcal bacteria is consistent with these symptoms^10^. Our study also found elevated levels of *Bifidobacterium* in the nares microbiome during spaceflight. Oral administration of some *Bifidobacterium* species has been shown to ameliorate allergic rhinitis symptoms in human patients^70^ and in a mouse model of allergic rhinitis^71^. Whether the observed increase of *Bifidobacterium* abundance in the nares may have a similar protective effect on astronauts during spaceflight warrants further investigations.

Our data show that inflight compositional changes in the GI, nose and skin microbiomes occur very early during the space mission and in most astronauts are readily evident by FD7. In addition, we found that these changes remain relatively stable in space, at least for up to 180 days.

It is known that stress may have a deleterious effect on the human microbiome and the immune response^47,72–75^. In this study we measured latent herpes virus reactivation and the concentration of stress hormones in saliva, markers frequently used to assess astronaut stress during space missions^12,13^. This analysis found four astronauts with significantly elevated levels of salivary VZV DNA by FD90 compared to preflight samples. Also, our analysis did not find any associations between EBV or HSV1 virus reactivation and space travel. However, of the three astronauts positive for EBV, two had elevated levels of EBV DNA in saliva during space flight and the remaining astronaut shed VZV in space. These results are consistent with previous studies showing that astronauts with latent EBV infections often experience viral reactivation well before their departure to space and many remain positive after their return to Earth^13^. Also, our assessment of VZV DNA in saliva agrees with studies showing that VZV reactivation in astronauts occurs mainly during the space mission and continues after astronauts return to Earth^76^. It is yet not clear the medical implications of latent virus reactivation during space flight, considering that none of the participating subjects had episodes of mononucleosis or reported the appearance of blisters and therefore, the observed reactivations were subclinical.

Our analysis also detected significant changes associated with space flight in two out of the three stress hormones measured. Salivary alpha-amylase, a marker associated with psychosocial stress^77^, was found elevated after the second half of the space mission. Alpha-amylase has been also proposed as a positive surrogate marker of activity of the sympathetic nervous system. Therefore, the elevated levels of alpha-amylase could be reflective of the increased sympathetic activity experienced by astronauts in space^78^. In addition, our study found that DHEA concentration in saliva was lower than the preflight baseline starting at FD90 and tended to remain low up to 180 days after the return to Earth, as it has been previously reported for other space missions^12^. Studies have shown that DHEA levels are increased by acute stress and that may have a beneficial effect on helping people cope with unwanted neurobehavioral effects caused by stress^79^. DHEA has also been shown to have a protective effect against a number of infections in mice, including viral infections such as herpes simplex type 2^80,81^ and play a role in the regulation of the immune response during acute stress^82^. Whether the inflight reduction in DHEA contributes to VZV and EBV reactivation during space missions warrants further investigations.

Our study did not detect any significant change in the salivary concentration of cortisol during flight, even though previous studies have found that this hormone increase during a mission to space^13^. One possible explanation of this discrepancy is that salivary cortisol levels start high early in the morning and rapidly decrease during the day. Participating crewmembers collected their saliva samples at the beginning of the day, but the specific time of collection was not fixed. Therefore, it is possible that the noise introduced in the data by shifts in saliva collection times plus the low number of participating subjects reduced the detection power of our assay. However, we cannot rule out other potential factors.

Examination of six distinct surfaces of the ISS revealed that there is a strong interaction between the forehead and forearm skin microbiome of astronaut crew members and the ISS environments surveyed in our study. Studies of the environmental microbiome of family homes^83^ and sterile rooms^84^ have demonstrated the rapid transfer of skin associated microbes from individuals to the areas they reside in. Also, a previous 16S rRNA gene survey of the ISS environment^85^ observed an abundance of human skin associated microbes throughout the ISS but was not able to compare those samples to the skin microbes of the astronauts residing at ISS at the time.

The ISS is an extremely isolated environment with a low influx of new microbes, so it is not surprising that the surfaces of ISS resemble astronaut skin microbiomes. It is interesting to note that most microbes detected on surfaces within the ISS are transient, and that shortly after one crew departs and a new crew arrives, the distribution of microbes on the ISS changes to reflect its new crew’s skin microbiome. It is also important to note that samples taken from ISS surfaces later in the study no longer had any similarity to earlier crew member skin microbiomes. Still, we found that a small proportion of the environmental bacteria were ubiquitously present in the ISS and therefore, are likely either long-term residents of the space station or microbes that are extremely common on humans. These results agree with a previous 16S rRNA gene survey that showed that the bacterial communities of the ISS mostly belonged to the phyla Actinobacteria, Proteobacteria and Firmicutes^85^. The same study also found that *Corynebacterium* and *Staphylococcus* were common in the ISS. In addition to these two genera, our study also found a high prevalence of species of the genera *Streptococcus* and *Cloacibacterium* on ISS surfaces. Although many species of *Corynebacterium*, *Staphylococcus*, *Streptococcus* and *Cloacibacterium* are non-pathogenic natural commensals of the human skin and mucosa, there are a few species that are considered opportunistic pathogens that lead to a number of human infections^86–90^. Nevertheless, the virulence characteristics of these bacterial species need to be further investigated before evaluating their potential risk to astronauts’ health during long-duration space missions. It is noteworthy that even though highly prevalent residents of the ISS represented less than 4% of the OTUs identified on ISS surfaces, more than 65% of them had their abundance differentially changed on the two skin sites. It is not clear why there was an overrepresentation of Beta and Gammaproteobacteria species in the ISS while the abundance of the same bacterial OTUs drops on the astronauts’ skin. One plausible explanation could be that potential changes in the skin structure during long stays in space^61^ enhances the translocation of Beta and Gamma proteobacteria from the crew’s skin to the ISS environment. The accompanying increase in the relative abundance of skin *Actinobacteria* and *Bacilli* could then be the direct result of the drop of skin Proteobacteria or due to factors associated with the ISS environment that favor their growth or prolong their survivability.

It should be noted that general trends described in this paper do not necessarily reflect what happens at the single subject level. For example, although *Akkermansia* abundance drops in inflight stool samples of all 5 astronauts, this change is only statistically significant in two out of the five astronauts. This could be due to a drop in statistical power when the analysis is done at the individual level or could indicate that the differences are actually reflective of individuals having different reactions to spaceflight based on a range of factors like their own personal chemistry or diet habits. Studies involving a far larger number of individuals will be required to determine global patterns of microbiome change to spaceflight, model microbiome responses in space to enable prediction of changes likely for an individual and differentiate benign microbiome perturbations from those that are likely to negatively impact any given astronaut’s health.

## Methods

### Human ethics statement

Subjects provided written, informed consent prior to study enrolment. The Institutional Review Boards from NASA (IRB protocol Pro0316) and JCVI (IRB protocol JCVI-2012-158) approved the study, which complied with the Declaration of Helsinki and good clinical practice, including data monitoring.

### Astronaut recruitment (inclusion/exclusion criteria)

Nine astronauts participated in this study and were designated A-I to provide the participants with as much anonymity as possible. While many identifying characteristics of each astronaut (e.g. profession, age, nationality) are not included in this work to provide as much participant privacy as possible, the information was provided to the authors to look for correlations based on these factors. Astronauts that participated in this study spent 6 months at the International Space Station, except for one individual that spent 1-year, and participants were given the option to not collect stool samples in-flight to ensure sufficient participation. Of the nine participants, four opted out of in-flight stool collection. Two of the astronauts were women, and seven were men. Astronauts participate in many studies while on earth or at ISS, and astronauts participating in studies that would influence or manipulate their microbiome (e.g. taking a probiotic) directly or indirectly were not eligible. Participants were all adults and had varied professional careers. All were considered healthy individuals for their gender and age. Some of the astronauts had previously spent time at ISS, while for others, this was their first mission to space.

### Sampling

#### Timing of sample collection

Astronauts collected longitudinal samples of their own microbiome, at 10 time points (Fig. 1). Because spaceflight is dictated by factors outside the control of the participants of the study, the sampling times are all related to two important change events. The first event is launch (to the ISS) and sample time points from before this point are designated L- and the number of days (e.g. L-240 indicates a sample was taken 240 days before launch). The second change event is their return from ISS to Earth, and samples taken after they return from ISS are designated R− and R+ and the number of days before or after the return. The samples taken during spaceflight are designated FD (Flight Day) and the number of days spent in the ISS at the time of sample collection.

#### Astronaut samples and experimental design

This is a longitudinal study. There is no true positive control for a human microbiome due to high variability between individuals, so sampling began 240 days before flight (L-240) to establish a baseline of microbiome variability and content. Four samples (L-240, L-150, L-90, and L-60) were taken pre-flight. Three in-flight samples (FD7, FD90 and FD180) were collected for nine of the astronauts. An additional FD360 time point was taken by the 1-year astronaut. Three samples (R + 0/3, R + 30 and R + 60) were collected post flight, with R + 0/3 designating the first sample collected after returning to Earth (the precise timing of this sample varied between individuals). All samples were self-collected by the astronauts, based on a standardized procedure and briefing by NASA personnel using a provided sampling kit.

Each astronaut collected a range of samples at each time point. During pre- and post-flight time points, they collected five personal microbiome swabs (forehead, both forearms, interior nares, tongue and stool) at each time point. While at ISS, astronauts took each of these samples, plus environmental swabs at six locations on ISS (ssA, the ARED Handle Bar; ssB, Intermodular Ventilation -IMV- Inlet; ssC, Cupola Nadir Window Shade Knob; ssD, Crew Quarters Stationary Light Knob; ssE, Smoke Detector; ssF, Handheld Microphone handle/grip). For the periods when two astronauts were concurrently at ISS, only a single set of environmental swabs was taken. Blood samples for cytokine analysis were taken at five time points (L-60, FD7, R-1, R + 0/1 and R + 180) and saliva samples for viral reactivation analysis were taken at the same times as microbiome samples, plus an R + 180 sample.

Samples taken on Earth were stored at −80 °C, while samples collected on ISS were stored at −100 °C until shipped to Earth.

### Cytokines

#### Measurement of cytokines in plasma

The concentrations for 22 plasma cytokines representing five broad categories of function (Table S1) were determined simultaneously in duplicate using a commercially available magnetic multiplex bead immunoassay (R&D Systems). Samples were processed according to the manufacturer’s instructions. Briefly, 50 μl of plasma were incubated with beads bound to a cytokine capture antibody. The 22 bead populations vary by fluorescence intensity so that they may be resolved for individual analysis. Bead cytokine concentrations were then washed and incubated with a fluorescent secondary antibody, specific for each cytokine, but fluorescing along a single channel distinct from the bead populations. The assay was performed in a 96 well plate, and analysis was performed using a Luminex Magpix instrument (Luminex, Inc., Austin, Texas). Samples from a single crewmember were processed in a single batch.

#### Statistical analysis of cytokine profiles

Inference on the dynamic effect of flight on cytokine expression levels was based on regression models for 19 of the 22 cytokines originally considered. For 17 of these cytokines, expression values were transformed to satisfy statistical model assumptions for mixed-effects regression such as normality and homoscedasticity, however, the particular transformation used depended on the cytokine (Table S2). In addition, expression values for five cytokines were sometimes below detectable limits (left censoring). For two of these five, we were still able to compare the time periods using right-censored lognormal regression on the reciprocal expression levels; however, there were too many censored observations to allow analysis for the remaining three cytokines (Table S2). For each of the 19 cytokines analyzed, we considered four comparisons (FD7, FD180, R + 0, and R + 180) with respect to the baseline time point at L-60. Thus overall, we made 76 tests of the null hypothesis of no effect. To correct for multiplicity, statistical significance was defined as p-values being less than a threshold calculated by controlling the false-discovery rate (FDR) to an acceptable level using the method of Benjamini and Hochberg (82) as implemented in Stata 15 software (StataCorp. 2017. Stata Statistical Software: Release 15. College Station, TX: Stata Corp LP). For reporting purposes, we used the threshold p < 0.0063 corresponding to a FDR of 5%, However the level of FDR necessary to consider a particular test result and all others with smaller p-values significant, known as the “q”-value was also calculated and included in Table S3.

#### Exploring possible association between changes in cytokine expression and bacteria relative abundance

In an exploratory analysis, we used Somers’ D to quantify relations between expression levels for each of 22 cytokines in combination with relative abundance for each of 32 OTUs found to undergo change during spaceflight (FDR ≤ 0.1) in the GI. Somers’ D is a non-parametric measure of association related to Kendall’s Tau. Kendall’s Tau can be expressed as the probability of “concordance” minus the probability of “discordance” between randomly selected pairs taken from two disparate but quantitative measures “*Y*” and “*X*”. In this application, “*Y*” is the bacterial relative abundance and “*X*” is the cytokine expression level. Because we are interested in comparing changes within subjects the pairs were restricted to the same subject’s test sessions comprising L-60, FD7, FD180, R + 0, and R + 180. Only data from stool and blood samples that were taken at approximately these times were used in this analysis. In the definition of Tau, “concordance” means that the change in relative abundance from one session to another and the corresponding change in expression level were in the same direction. “Discordance” means that the changes were in opposite directions. Somers’ D is defined similarly, except that the probabilities are conditional, excluding ties in the first measure (relative abundance). This is appropriate in this application because we only considered changes in relative abundance that were at least two-fold. Being a difference of two probabilities, Somers’ D is scaled within the range of −1 (perfect discordance) to +1 (perfect concordance) between the two variables of interest.

To implement the analysis, we first transformed relative abundance (*RA*) to discrete levels that differed by at least twofold by defining *Y* as the nearest multiple of log(2) in log(*RA*) for *RA* > 0. For the case *RA* = 0, we defined *Y* to be one multiple of log(2) less than the lowest level for *RA* > 0. By using *Y* instead of *RA* in the calculation of D, we ensured that only changes in *RA* of at least twofold are considered. Defining *Y* as above and *X* = the log transform of cytokine expression, we used the command *somersd* as implemented in the Stata 15 Statistical Software (Release 15) to calculate D, its standard error, and corresponding *p*-value for 704 = 32 × 22 combinations of bacteria and cytokines. To flag combinations as having shown “significant” association, we again used the method described in^91^ to control the false discovery rate (FDR) to 1%, corresponding to a *p*-value threshold of 0.00038. Because the resampling method used to obtain the standard error of D is not reliable for very small samples, we excluded from consideration any combination that failed to support at least 10 comparisons with RA > 0 and cytokine expression above the lowest detectable limit. As a result, *p*-values were actually calculated for only 487 of the original 704 combinations.

### Reactivated viruses & stress hormones

#### Saliva sample collection

Saliva samples for viral assessments were collected using Salivette synthetic rolls (Salimetrics, LLC., PA) immediately after their sleep cycle, before eating and brushing their teeth. To collect a sample, the subject placed the synthetic roll in their mouth until it became saturated with saliva (2–3 minutes). The saturated Salivette was then stored frozen at −80 °C until processed^12,92^. Four saliva samples were collected at each of 10 sampling sessions, as described above. Upon return to Earth, flight saliva samples collected via Salivettes were centrifuged to separate fluid from the swab, and the supernatant fluid was stored frozen (−80 °C) until processed.

#### Detection of Viral DNA in saliva samples

Viral DNA was extracted by a nonorganic extraction method (Qiagen Inc., Chatsworth, CA) QIAamp Viral RNA Kits (Qiagen Inc., Santa Clarita, CA) were used to extract viral genomic DNA from concentrated saliva and urine, and each assay was performed according to manufacturer’s instructions. HSV1, EBV, and VZV DNA were measured in saliva by real-time PCR using an ABI 7900 (Applied Biosystems, Foster City, CA) PCR system. The primers and probes used for EBV and VZV have been published previously^12,92^.

#### Salivary cortisol, DHEA and amylase

Saliva samples collected as explained above were also processed for measuring cortisol, DHEA and amylase by standard ELISA method by using kits from Salimetrics as previously described. Intra and inter-assay coefficients of variation for cortisol and DHEA were less than 5% and 10%, respectively using this procedure.

To test if the inflight/postflight frequency of detection of latent virus in saliva or the concentration of stress hormones were significantly different from preflight baseline values, a linear mixed-effects model was applied with *mission time point* as the fixed-effect term (using the pooled preflight values as baseline) and *astronaut* as the random-effect term with only random intercept.

### DNA sequencing

#### DNA extraction and 16S rRNA gene profiling by MiSeq sequencing

Stool samples were extracted using the Qiagen DNeasy Powersoil Kit using manufacturer’s specifications. DNA from environmental and body sample swabs were resuspended in 1,200 μl of lysis buffer (20 mM Tris-Cl, pH 8.0, 2 mM EDTA, 1.2% Triton X-100). Sample tubes containing swab and buffer were vortexed thoroughly at maximum speed for 1 minute. 1,000 μl of lysis buffer from the sample tube containing the original swab sample was removed and placed into a lysing Matrix B tube (MP Biomedicals Cat # 6911-500, http://www.mpbio.com/). Matrix tubes were then vortexed at maximum speed for 5 minutes followed by centrifugation at 10,000 RPM for 1 minute. 700 μl of lysate from the Matrix B tube was removed and incubated at 75 °C for 10 minutes. When cooled to room temperature the lysate was then treated with 200 mg/ml lysozyme and 20 mg/ml Proteinase K. DNA from the lysate was twice extracted using Phenol Chloroform Isoamyl Alcohol followed by ethanol precipitation. DNA extracted from stool and swab samples was amplified using primers that targeted the V4 region of the 16S rRNA gene^93,94^. These primers included the i5 and i7 adaptor sequences for dual index Illumina MiSeq as well as unique 8 bp indices incorporated onto both primers such that each sample received its own unique barcode pair. Using approximately 100 ng of extracted DNA, the amplicons were generated with Platinum Taq polymerase (Life Technologies, CA) and purified using the QIAquick PCR purification kit (Qiagen Valencia, CA). Amplicons were then quantified using Tecan fluorometric methods (Tecan Group Mannedorf, Switzerland), normalized, and then pooled in preparation for Illumina MiSeq sequencing using the dual index V2 chemistry 2 × 250 bp format (Illumina, San Diego, CA) following the manufacturer’s protocol.

### Sequence Analysis

#### 16S rRNA gene Taxonomic Profiles

16S rRNA gene sequences from all samples were processed using an in-house pipeline based on the clustering algorithm UParse (usearch vrsion 8.1.1861)^95^ and Mothur (version 1.36.1)^96^. Briefly, read mates were first merged with usearch option -fastq_mergepairs and minimum fastq read length of 50 bp, and thereafter, merged sequences were further quality filtered with usearch options -fastq_filter and -fastq_maxee 1. Next, merged reads were dereplicated with usearch -derep_fulllength and sorted by abundance with usearch options -sortbysize and -minsize 2. 16S rRNA amplicon sequences were then clustered at 97% identity and chimeras filtered with usearch option -cluster_otus. Taxonomic classification was then performed on representative OTU cluster sequences with the classify.seqs function from mothur with options iters = 100 and cutoff = 80 against the SILVA SSU Ref NR99 database (version 123)^97^. Finally, OUT count tables were generated with usearch option -usearch_global with the following parameters: -id 0.97, -otutabout, -biomout and -mothur_shared_out. Taxonomic and statistical analyses were performed in R (version 3.5). OTUs were required to be present in at least 4 samples with at least 4 reads per sample. Fourteen OTUs that were supported by 5 or more reads in either the PCR negative controls or DNA extraction kit control samples were removed from the analysis. The R function *decostand* (Vegan) was used to correct for variable read depth per sample.

#### Analysis of alpha diversity and richness

Shannon alpha diversity and Richness indexes were estimated with the *diversity* and *rarefy* functions from the R package Vegan^98^. Inference on the effect of space flight on the alpha diversity and Richness of the different microbial communities surveyed was based on linear mixed-effects models using the R packages *lme4*^99^ and *lmerTest*^100^ with the categorical variable *mission stage* as the fixed-effect term (using “Preflight” as the baseline level) and *astronaut* as the random-effect term with only random intercept.

Pearson correlation analyses of either Shannon alpha diversity or Richness values between skin and ISS-surface samples collected at FD7 and FD180 (Fig. 8) were carried out with the R function stat_cor of the package *ggpubr*.

#### Analysis of Bray-Curtis beta diversity

To compare taxonomic profiles across microbiome samples, weighted and unweighted Bray-Curtis beta diversity distances were calculated across all human and ISS samples collected during the study with the R function *vegdist* (Vegan) with the following parameters: method = ”bray” for weighted Bray-Curtis beta diversity, or method = ”bray” binary = TRUE for unweighted Bray-Curtis beta diversity. Compositional differences among samples from different body and ISS sites (Figs 2A, 7A,B, S1A, S3 and S10) were evaluated with Permutational Multivariate Analysis of Variance (PERMANOVA) with the R function *adonis* (Vegan), while microbial compositional changes of samples collected from the same (within) or different (between) astronauts (Fig. S2) were assessed by comparing their mean Bray-Curtis distances using Welch two-sample t-tests.

To investigate the impact of space flight on the astronauts’ microbiota at each body site (Fig. 4), differences in the mean Bray-Curtis distance within and between mission stages (preflight, inflight and postflight) were evaluated applying a linear mixed-effects model design with the category within/between stages as the fixed-effect term and categorical variable *astronaut* as the random-effect term with only random intercept. Bray-Curtis distances between samples collected from different astronauts were eliminated from the analysis. In addition, for each body site, assessment of the impact of time spent at the ISS on the crew microbiota was performed by comparing the mean Bray-Curtis distance among samples collected at all preflight time points, used as the baseline, and the mean Bray-Curtis distance between samples collected at every inflight/postflight timepoint and every preflight timepoint. As previously described, only within-astronaut distances were considered for the analysis. Statistical significance was evaluated using linear mixed-effects models with mission time point as the fixed-effect term and categorical variable *astronaut* as the random-effect term with only random intercept.

To investigate whether the astronauts’ GI microbiome becomes more similar in the ISS, the mean inter-astronaut Bray-Curtis distances were compared between preflight samples and inflight or postflight samples using a Welch two-samples t-test (Fig. S7B). Likewise, changes in the mean BC distances between skin and ISS samples collected at either FD7 or FD180 time points during space flight (Fig. 7C,D) were assessed using a Welch two-samples t-test.

#### Determination of astronauts’ preflight core microbiomes and identification of phylotypes whose relative abundance change during the mission

For each of the five human sites sampled a core microbiome was defined as all genera or OTUs that were present in at least 75% of all preflight samples collected for the study and that were supported by at least four reads per sample. Differential phylotype abundance analysis between preflight samples, as baseline, and either inflight or postflight samples were performed with the R packages EdgeR^101^ and DESeq2^102^. using a design matrix derived from the additive model formula “~astronaut + mission_stage” to account for crew-specific changes^101^. Resulting p-values were then adjusted for multiple testing using the false discovery rate method. Taxa with a FDR < 0.05 for both methods were considered significant.

Phylotypes that could potentially be permanent residents of each of the six ISS surfaces surveyed were identified using an in-house R script that identified OTUs that were detected at every relative time point during the study and whose relative abundance was not zero for two or more consecutive relative time points.

## Supplementary information

Supplementary information

Supplementary Table S1

Supplementary Table S2

Supplementary Table S3

Supplementary Table S4

Supplementary Table S5

Supplementary Table S6

Supplementary Table S7

Supplementary Table S8

## Data Availability

All data generated or analyzed during this study are included in this published article and its Supplementary Information files. 16S rRNA gene sequencing reads used in this study are available at NASA Life Sciences Data Archive (LSDA) repository (https://lsda.jsc.nasa.gov).

## References

[1] Ohnishi K, Ohnishi T (2004). The biological effects of space radiation during long stays in space. Biol Sci Space.

[2] Mallis MM, DeRoshia CW (2005). Circadian rhythms, sleep, and performance in space. Aviat Space Environ Med.

[3] Crucian BE (2018). Immune System Dysregulation During Spaceflight: Potential Countermeasures for Deep Space Exploration Missions. Front Immunol.

[4] Hackney KJ (2015). The Astronaut-Athlete: Optimizing Human Performance in Space. J Strength Cond Res.

[5] Wu B (2018). On-orbit sleep problems of astronauts and countermeasures. Mil Med Res.

[6] Zhang LF, Hargens AR (2018). Spaceflight-Induced Intracranial Hypertension and Visual Impairment: Pathophysiology and Countermeasures. Physiol Rev.

[7] *NASA Human Research Roadmap*, https://humanresearchroadmap.nasa.gov/explore/ (2019).

[8] Howe Alexis, Kiffer Frederico, Alexander Tyler C., Sridharan Vijayalakshmi, Wang Jing, Ntagwabira Fabio, Rodriguez Analiz, Boerma Marjan, Allen Antiño R. (2019). Long-Term Changes in Cognition and Physiology after Low-Dose 16O Irradiation. International Journal of Molecular Sciences.

[9] Bigley AB (2019). NK cell function is impaired during long-duration spaceflight. J Appl Physiol (1985).

[10] Crucian B (2016). Incidence of clinical symptoms during long-duration orbital spaceflight. Int J Gen Med.

[11] Antonsen, E. *Risk of Adverse Health Outcomes & Decrements in Performance due to Inflight Medical Conditions*, https://humanresearchroadmap.nasa.gov/Risks/risk.aspx?i=95 (2017).

[12] Mehta SK (2014). Multiple latent viruses reactivate in astronauts during Space Shuttle missions. Brain Behav Immun.

[13] Pierson DL, Stowe RP, Phillips TM, Lugg DJ, Mehta SK (2005). Epstein-Barr virus shedding by astronauts during space flight. Brain Behav Immun.

[14] Crucian B (2015). Alterations in adaptive immunity persist during long-duration spaceflight. NPJ Microgravity.

[15] Crucian BE, Stowe RP, Pierson DL, Sams CF (2008). Immune system dysregulation following short- vs long-duration spaceflight. Aviat Space Environ Med.

[16] Kaur I, Simons ER, Castro VA, Mark Ott C, Pierson DL (2004). Changes in neutrophil functions in astronauts. Brain Behav Immun.

[17] Stowe RP, Sams CF, Pierson DL (2003). Effects of mission duration on neuroimmune responses in astronauts. Aviat Space Environ Med.

[18] Crucian B (2016). A case of persistent skin rash and rhinitis with immune system dysregulation onboard the International Space Station. J Allergy Clin Immunol Pract.

[19] Li P (2015). Simulated microgravity disrupts intestinal homeostasis and increases colitis susceptibility. FASEB J.

[20] Foster JS, Khodadad CL, Ahrendt SR, Parrish ML (2013). Impact of simulated microgravity on the normal developmental time line of an animal-bacteria symbiosis. Sci Rep.

[21] Nickerson CA, Ott CM, Wilson JW, Ramamurthy R, Pierson DL (2004). Microbial responses to microgravity and other low-shear environments. Microbiol Mol Biol Rev.

[22] Pierson DL (1996). Epidemiology of Staphylococcus aureus during space flight. FEMS Immunol Med Microbiol.

[23] Johnston, R. S. & Dietlein, L. F. Skylab Environmental and Crew Microbiology Studies. (NASA, 1977).

[24] Human Microbiome Project Consortium (2012). Structure, function and diversity of the healthy human microbiome. Nature.

[25] Costello EK (2009). Bacterial community variation in human body habitats across space and time. Science.

[26] Shao D (2017). Simulated microgravity affects some biological characteristics of Lactobacillus acidophilus. Appl Microbiol Biotechnol.

[27] Klaus DM, Howard HN (2006). Antibiotic efficacy and microbial virulence during space flight. Trends Biotechnol.

[28] Ciferri O, Tiboni O, Di Pasquale G, Orlandoni AM, Marchesi ML (1986). Effects of microgravity on genetic recombination in Escherichia coli. Naturwissenschaften.

[29] Benoit MR (2006). Microbial antibiotic production aboard the International Space Station. Appl Microbiol Biotechnol.

[30] Decelle JG, Taylor GR (1976). Autoflora in the upper respiratory tract of Apollo astronauts. Appl Environ Microbiol.

[31] Lencner AA (1984). The quantitative composition of the intestinal lactoflora before and after space flights of different lengths. Nahrung.

[32] Brown LR, Fromme WJ, Handler SF, Wheatcroft MG, Johnston DA (1976). Effect of Skylab missions on clinical and microbiologic aspects of oral health. J Am Dent Assoc.

[33] Lizko NN, Silov VM, Syrych GD (1984). Events in he development of dysbacteriosis of the intestines in man under extreme conditions. Nahrung.

[34] Nefedov YG, Shilov VM, Konstantinova IV, Zaloguyev SN (1971). Microbiological and immunological aspects of extended manned space flights. Life Sci Space Res.

[35] Taylor PW, Sommer AP (2005). Towards rational treatment of bacterial infections during extended space travel. Int J Antimicrob Agents.

[36] Hales, N. W. *et al*. A countermeasure to ameliorate immune dysfunction in *in vitro* simulated microgravity environment: role of cellularnucleotide nutrition. *In Vitro Cell Dev Biol Anim* **38**, 213–217, doi:10.1290/1071-2690(2002)038<0213:ACTAID>2.0.CO;2 (2002). 10.1290/1071-2690(2002)038<0213:ACTAID>2.0.CO;2 12197773

[37] Mosca A, Leclerc M (2016). & Hugot, J. P. Gut Microbiota Diversity and Human Diseases: Should We Reintroduce Key Predators in Our Ecosystem?. Front Microbiol.

[38] Ritchie LE (2015). Space Environmental Factor Impacts upon Murine Colon Microbiota and Mucosal Homeostasis. PLoS One.

[39] Qin J (2010). A human gut microbial gene catalogue established by metagenomic sequencing. Nature.

[40] Miquel S (2013). Faecalibacterium prausnitzii and human intestinal health. Curr Opin Microbiol.

[41] Lopez-Siles M, Duncan SH, Garcia-Gil LJ, Martinez-Medina M (2017). Faecalibacterium prausnitzii: from microbiology to diagnostics and prognostics. ISME J.

[42] Crucian BE (2014). Plasma cytokine concentrations indicate that *in vivo* hormonal regulation of immunity is altered during long-duration spaceflight. J Interferon Cytokine Res.

[43] Strewe C (2012). Effects of parabolic flight and spaceflight on the endocannabinoid system in humans. Rev Neurosci.

[44] Chen YJ (2018). Parasutterella, in association with irritable bowel syndrome and intestinal chronic inflammation. J Gastroenterol Hepatol.

[45] Takeshita K (2016). A Single Species of Clostridium Subcluster XIVa Decreased in Ulcerative Colitis Patients. Inflamm Bowel Dis.

[46] Henning SM (2018). Decaffeinated green and black tea polyphenols decrease weight gain and alter microbiome populations and function in diet-induced obese mice. Eur J Nutr.

[47] Bailey MT (2011). Exposure to a social stressor alters the structure of the intestinal microbiota: implications for stressor-induced immunomodulation. Brain Behav Immun.

[48] Gabay C (2006). Interleukin-6 and chronic inflammation. Arthritis Res Ther.

[49] Li B (2015). IL-10 engages macrophages to shift Th17 cytokine dependency and pathogenicity during T-cell-mediated colitis. Nat Commun.

[50] McGeachy MJ, Cua DJ (2008). Th17 cell differentiation: the long and winding road. Immunity.

[51] Scher JU (2015). Decreased bacterial diversity characterizes the altered gut microbiota in patients with psoriatic arthritis, resembling dysbiosis in inflammatory bowel disease. Arthritis Rheumatol.

[52] Dao MC (2016). Akkermansia muciniphila and improved metabolic health during a dietary intervention in obesity: relationship with gut microbiome richness and ecology. Gut.

[53] Png CW (2010). Mucolytic bacteria with increased prevalence in IBD mucosa augment *in vitro* utilization of mucin by other bacteria. Am J Gastroenterol.

[54] Vigsnaes LK, Brynskov J, Steenholdt C, Wilcks A, Licht TR (2012). Gram-negative bacteria account for main differences between faecal microbiota from patients with ulcerative colitis and healthy controls. Benef Microbes.

[55] Rajilic-Stojanovic M, Shanahan F, Guarner F, de Vos WM (2013). Phylogenetic analysis of dysbiosis in ulcerative colitis during remission. Inflamm Bowel Dis.

[56] Ottman, N. *et al*. Genome-Scale Model and Omics Analysis of Metabolic Capacities of Akkermansia muciniphila Reveal a Preferential Mucin-Degrading Lifestyle. *Appl Environ Microbiol* **83**, 10.1128/AEM.01014-17 (2017). 10.1128/AEM.01014-17 PMC5583483 28687644

[57] Cani PD (2016). Endocannabinoids–at the crossroads between the gut microbiota and host metabolism. Nat Rev Endocrinol.

[58] Ruokolainen L (2015). Green areas around homes reduce atopic sensitization in children. Allergy.

[59] Hanski I (2012). Environmental biodiversity, human microbiota, and allergy are interrelated. Proc Natl Acad Sci USA.

[60] Fyhrquist N (2014). Acinetobacter species in the skin microbiota protect against allergic sensitization and inflammation. J Allergy Clin Immunol.

[61] Tronnier H, Wiebusch M, Heinrich U (2008). Change in skin physiological parameters in space–report on and results of the first study on man. Skin Pharmacol Physiol.

[62] Zeeuwen PL (2012). Microbiome dynamics of human epidermis following skin barrier disruption. Genome Biol.

[63] Musher DM (1994). The current spectrum of Staphylococcus aureus infection in a tertiary care hospital. Medicine (Baltimore).

[64] Sharafutdinov IS (2017). Antimicrobial Effects of Sulfonyl Derivative of 2(5H)-Furanone against Planktonic and Biofilm Associated Methicillin-Resistant and -Susceptible Staphylococcus aureus. Front Microbiol.

[65] Cannon JW (2018). An economic case for a vaccine to prevent group A streptococcus skin infections. Vaccine.

[66] Feazel LM, Robertson CE, Ramakrishnan VR, Frank DN (2012). Microbiome complexity and Staphylococcus aureus in chronic rhinosinusitis. Laryngoscope.

[67] Ramakrishnan VR, Feazel LM, Abrass LJ, Frank DN (2013). Prevalence and abundance of Staphylococcus aureus in the middle meatus of patients with chronic rhinosinusitis, nasal polyps, and asthma. Int Forum Allergy Rhinol.

[68] Jervis Bardy J, Psaltis AJ (2016). Next Generation Sequencing and the Microbiome of Chronic Rhinosinusitis: A Primer for Clinicians and Review of Current Research, Its Limitations, and Future Directions. Ann Otol Rhinol Laryngol.

[69] Muluk NB, Altin F, Cingi C (2018). Role of Superantigens in Allergic Inflammation: Their Relationship to Allergic Rhinitis, Chronic Rhinosinusitis, Asthma, and Atopic Dermatitis. Am J Rhinol Allergy.

[70] Singh A (2013). Immune-modulatory effect of probiotic Bifidobacterium lactis NCC2818 in individuals suffering from seasonal allergic rhinitis to grass pollen: an exploratory, randomized, placebo-controlled clinical trial. Eur J Clin Nutr.

[71] Ren J (2018). Immunomodulatory effect of Bifidobacterium breve on experimental allergic rhinitis in BALB/c mice. Exp Ther Med.

[72] Bailey MT (2014). Influence of stressor-induced nervous system activation on the intestinal microbiota and the importance for immunomodulation. Adv Exp Med Biol.

[73] Bailey MT, Engler H, Sheridan JF (2006). Stress induces the translocation of cutaneous and gastrointestinal microflora to secondary lymphoid organs of C57BL/6 mice. J Neuroimmunol.

[74] De Palma G (2015). Microbiota and host determinants of behavioural phenotype in maternally separated mice. Nat Commun.

[75] Rea K, Dinan TG, Cryan JF (2016). The microbiome: A key regulator of stress and neuroinflammation. Neurobiol Stress.

[76] Mehta SK (2004). Stress-induced subclinical reactivation of varicella zoster virus in astronauts. J Med Virol.

[77] Petrakova L (2015). Psychosocial Stress Increases Salivary Alpha-Amylase Activity Independently from Plasma Noradrenaline Levels. PLoS One.

[78] Mandsager KT, Robertson D, Diedrich A (2015). The function of the autonomic nervous system during spaceflight. Clin Auton Res.

[79] Morgan CA, Rasmusson A, Pietrzak RH, Coric V, Southwick SM (2009). Relationships among plasma dehydroepiandrosterone and dehydroepiandrosterone sulfate, cortisol, symptoms of dissociation, and objective performance in humans exposed to underwater navigation stress. Biol Psychiatry.

[80] Loria RM, Inge TH, Cook SS, Szakal AK, Regelson W (1988). Protection against acute lethal viral infections with the native steroid dehydroepiandrosterone (DHEA). J Med Virol.

[81] Loria RM, Padgett DA (1998). Control of the immune response by DHEA and its metabolites. Rinsho Byori.

[82] Prall SP, Larson EE, Muehlenbein MP (2017). The role of dehydroepiandrosterone on functional innate immune responses to acute stress. Stress Health.

[83] Lax S (2014). Longitudinal analysis of microbial interaction between humans and the indoor environment. Science.

[84] Meadow JF (2015). Humans differ in their personal microbial cloud. PeerJ.

[85] Checinska A (2015). Microbiomes of the dust particles collected from the International Space Station and Spacecraft Assembly Facilities. Microbiome.

[86] Leung TN, Hon KL, Leung AK, Group A (2018). Streptococcus disease in Hong Kong children: an overview. Hong Kong Med J.

[87] Rosa-Fraile M, Spellerberg B (2017). Reliable Detection of Group B Streptococcus in the Clinical Laboratory. J Clin Microbiol.

[88] Paharik, A. E. & Horswill, A. R. The Staphylococcal Biofilm: Adhesins, Regulation, and Host Response. *Microbiol Spectr* **4**, 10.1128/microbiolspec.VMBF-0022-2015 (2016). 10.1128/microbiolspec.VMBF-0022-2015 PMC4887152 27227309

[89] Bernard K (2012). The genus corynebacterium and other medically relevant coryneform-like bacteria. J Clin Microbiol.

[90] Mubaiwa, T. D. *et al*. The sweet side of the pathogenic Neisseria: the role of glycan interactions in colonisation and disease. *Pathog Dis* **75**, 10.1093/femspd/ftx063 (2017). 10.1093/femspd/ftx063 PMC5808653 28633281

[91] Benjamini Y, Krieger AM, Yekutieli D (2006). Adaptive linear step-up procedures that control the false discovery rate. Biometrika.

[92] Mehta SK (2017). Latent virus reactivation in astronauts on the international space station. NPJ Microgravity.

[93] Jeraldo P, Chia N, Goldenfeld N (2011). On the suitability of short reads of 16S rRNA for phylogeny-based analyses in environmental surveys. Environ Microbiol.

[94] Bokulich NA, Mills DA (2013). Improved selection of internal transcribed spacer-specific primers enables quantitative, ultra-high-throughput profiling of fungal communities. Appl Environ Microbiol.

[95] Edgar RC (2013). UPARSE: highly accurate OTU sequences from microbial amplicon reads. Nat Methods.

[96] Schloss PD (2009). Introducing mothur: open-source, platform-independent, community-supported software for describing and comparing microbial communities. Appl Environ Microbiol.

[97] Quast C (2013). The SILVA ribosomal RNA gene database project: improved data processing and web-based tools. Nucleic Acids Res.

[98] Dixon P (2003). VEGAN, a package of R functions for community ecology. J Veg Sci.

[99] Bates D, Mächler M, Bolker B, Walker S (2015). Fitting Linear Mixed-Effects Models Using. J Stat Softw.

[100] Kuznetsova, A., Brockhoff, P. B. & Christensen, R. H. B. lmerTest Package: Tests in Linear Mixed Effects Models. *J Stat Softw* **82**, 10.18637/jss.v082.i13 (2017).

[101] Robinson MD, McCarthy DJ, Smyth G (2010). K. edgeR: a Bioconductor package for differential expression analysis of digital gene expression data. Bioinformatics.

[102] Love MI, Huber W, Anders S (2014). Moderated estimation of fold change and dispersion for RNA-seq data with DESeq. 2. Genome Biol.

