# Supplementary material for: Study of the impact of long-duration space missions at the International Space Station on the astronaut microbiome
Source: Sci Rep. 2019 Jul 9;9:9911. doi: 10.1038/s41598-019-46303-8 (PMC6616552; doi:10.1038/s41598-019-46303-8)
Supplement: Supplementary file 1 — Supplementary information [file 41598_2019_46303_MOESM1_ESM.pdf]

Supplementary Information

Study of the impact of long-duration space missions at the International Space Station on the astronaut microbiome

Alexander A. Voorhies[1], C. Mark Ott[2], Satish Mehta[2], Duane L. Pierson[2], Brian E. Crucian[2], Alan Feiveson[2], Cherie M. Oubre[2], Manolito Torralba[1], Kelvin Moncera[1], Yun Zhang[1], Eduardo Zurek[4], and Hernan A. Lorenzi[1*].

1. Department of Infectious Diseases, J. Craig Venter Institute, Rockville MD, USA
2. NASA-Johnson Space Center, Houston TX, USA
3. Universidad del Norte, Barranquilla, Colombia

* Materials and corresponding author:
Email: hlorenzi@jcvi.org
The J. Craig Venter Institute
9605 Medical Center Drive
Suite 150
Rockville, MD 20850

Supplementary figure legends

Figure S1: principal coordinate plots of unweighted Bray-Curtis beta diversity distances across all samples collected in the study. A, samples coloured by body or ISS surface site. The permanova p-value using body site as the independent variable is shown. B, human microbiome samples coloured by astronaut identifier. Each microbiome is represented with a different symbol. Permanova p-value and R2 using astronaut identifier as the independent variable and stratifying by body site are indicated.

Figure S2: comparative analysis of beta diversity distances among microbial communities collected from the same or different crew members. Boxplot of weighted (A E) and unweighted (F - J) Bray-Curtis beta diversity distances between samples collected from the same (Intra) or different (Inter) astronauts. The Welch two-sample t-test p-values of the comparison of Inter- versus Intra-astronaut distances are indicated.

Figure S3: comparative beta diversity analysis among ISS microbial communities and nose, forehead and forearm skin microbiomes. Principal coordinate analysis of weighted (top panels) and unweighted (bottom panels) Bray-Curtis beta diversity among ISS environmental microbiota and forearm (A and D), forehead (B and E) and nose (C and F) microbiomes. Red circles, crew inflight samples; blue circles, crew pre and postflight samples; green circles, ISS environmental samples. Permanova p-values and R^2 values are indicated.

Figure S4: changes in alpha diversity and richness of individual crew members during a mission at the ISS. Boxplots depicting changes in Shannon alpha diversity (A) and Richness (B) of the five human microbiomes surveyed, by individual astronaut. Microbiome source is indicated on the right.

Figure S5: differential response of the astronauts' skin microbiota to the space environment. Boxplots depicting opposed responses of the skin microbiome to spaceflight. The forehead and forearm skin of astronauts A, B, D, F and H showed an increase in both Shannon alpha diversity (A) and species Richness (B) during spaceflight, while the same two skin sites of astronauts C, E, G and I responded in the opposite direction during spaceflight. Linear mixed-effects model p-values $\leqq 0.06$ are depicted using Preflight samples as a baseline.

Figure S6: compositional changes in the astronauts' microbiome during a space mission. Differences in the mean weighted (A) and unweighted (B) Bray-Curtis beta diversity distance between samples collected at individual inflight (blue) or postflight (yellow) time points and preflight samples. Grey boxplots represent the average within-astronaut weighted (A) or unweighted (B) Bray-Curtis distance among all preflight samples. Linear mixed-effects model p-values $\leqq 0.05$ are shown using within-preflight distances as baseline.

Figure S7: comparative analysis of inter-astronaut beta diversity distances of the GI microbiome before, during and after a space mission. A, Principal coordinate plot of weighted Bray-Curtis beta diversity distances among GI microbiome samples collected during preflight (blue) or inflight (red) stages. Astronauts B, C, D, F and G are represented with distinct symbols. B, boxplot showing that the weighted Bray-Curtis distance between GI samples from distinct crew members becomes smaller in the ISS compared to preflight samples. The Welch two-sample t-test p-values of the comparison between preflight distances versus inflight or postflight distances are shown.

Figure S8: changes in the relative abundance of bacterial genera of the astronauts' forehead skin during a mission at the ISS and after the return to Earth. Colours represent different phyla; horizontal axis, logarithm of the fold change in relative abundance between preflight and inflight or postflight samples; vertical axis, bacterial genera. Black circles indicate genera belonging to the corresponding preflight core microbiome.

Figure S9: bacterial genera of the GI microbiome that present the largest absolute difference between preflight and inflight coefficients of variation (CV) of their relative abundance across astronauts. Yellow bars indicate bacterial genera whose relative abundance becomes more similar in the ISS (CVpreflight >

CVinflight), while Blue bars represent those genera whose abundance becomes more different in space (CVpreflight < CVinflight).

Figure S10: changes in beta diversity of the environmental microbiome of the ISS. Principal coordinate plots of weighted (A and B) and unweighted (C and D) Bray-Curtis distances depicting shifts in the composition of the ISS microbiome across ISS surfaces (A and C) and over time (B and D). Panels A and C, distinct ISS surfaces are represented by different colours. Panels B and D, colours and numbers represent distinct relative mission time points, as indicated in Fig. 1B. Ellipses represent 95% confidence intervals. Permanova p-values < 0.05 and R^2 values are shown.

Figure S11: comparative analysis of alpha diversity and richness across inflight skin and ISS environmental samples. Boxplots depicting Shannon alpha diversity (A) and Richness (B) of inflight forehead and forearm skin samples and of the six ISS surfaces surveyed in this study.

Figure S12: bubble plot showing changes in the relative abundance of OTUs ubiquitously detected on ISS surface ssA during the entire study. Circle sizes are proportional to the relative abundance of each OTU at every relative time point. For each OTU, phylum and genus are indicated. No sample was collected at relative mission time 10.

Figure S13: bubble plot showing changes in the relative abundance of OTUs ubiquitously detected on ISS surface ssB during the entire study. Circle sizes are proportional to the relative abundance of each OTU at every relative time point. For each OTU, phylum and genus are indicated. No sample was collected at relative mission time 10.

Figure S14: bubble plot showing changes in the relative abundance of OTUs ubiquitously detected on ISS surface ssC during the entire study. Circle sizes are proportional to the relative abundance of each OTU at every relative time point. For each OTU, phylum and genus are indicated. No sample was collected at relative mission time 10.

Figure S15: bubble plot showing changes in the relative abundance of OTUs ubiquitously detected on ISS surface ssD during the entire study. Circle sizes are proportional to the relative abundance of each OTU at every relative time point. For each OTU, phylum and genus are indicated. No sample was collected at relative mission time 10.

Figure S16: bubble plot showing changes in the relative abundance of OTUs ubiquitously detected on ISS surface ssF during the entire study. Circle sizes are proportional to the relative abundance of each OTU at every relative time point. For each OTU, phylum and genus are indicated. No sample was collected at relative mission time 10.

Figure S17: bubble plot showing changes in the relative abundance of OTUs ubiquitously detected on the ISS surface ssE during the entire study. Circle sizes are proportional to the relative abundance of each OTU at every relative time point. For each OTU, phylum and genus are indicated. No sample was collected at relative mission time 10.

Figure S18: detection of viral DNA and stress hormones in saliva. A-C, qPCR detection of HSV1 (A), EBV (B) and VZV (C) genomic DNA in astronauts'' saliva during a mission at the ISS and after their return to Earth. Red line, mean viral DNA concentration in saliva across all virus-positive crewmembers. Dotted lines, viral DNA concentration levels for individual astronauts. D-F, changes in the concentration of alpha amylase (D), DHEA (E) and cortisol (F) in astronauts' saliva. Significance is depicted for mean values only. o, p-value < 0.1; *, p-value < 0.05; **, p-value < 0.01.

Figure S1

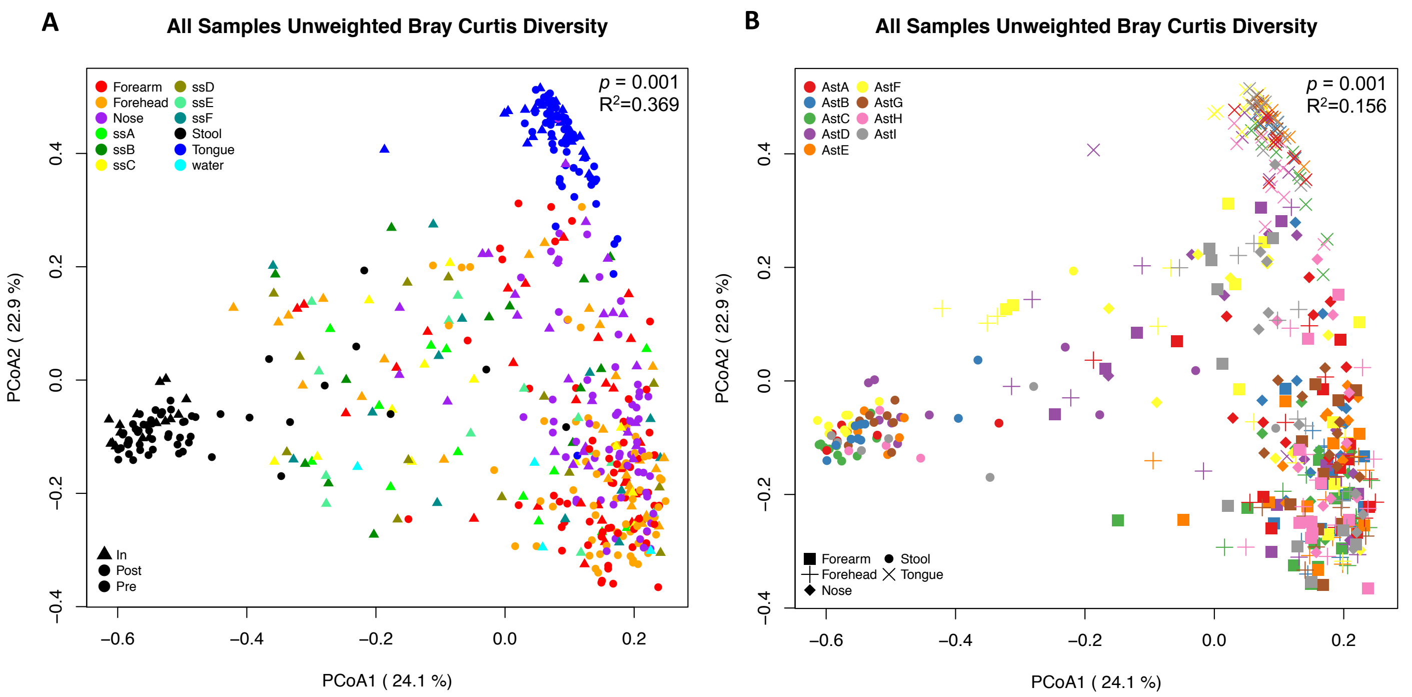
A
All Samples Unweighted Bray Curtis Diversity
Forearm
Forehead
Nose
ssA
ssB
ssC
ssD
ssE
ssF
Stool
Tongue
water
p = 0.001
R2=0.369
In
Post
Pre
PCoA1 (24.1 %)
PCoA2 (22.9 %)
B
All Samples Unweighted Bray Curtis Diversity
AstA
AstB
AstC
AstD
AstE
AstF
AstG
AstH
AstI
p = 0.001
R2=0.156
Forearm
Forehead
Nose
Stool
Tongue
PCoA1 (24.1 %)
PCoA2 (22.9 %)

Figure S2

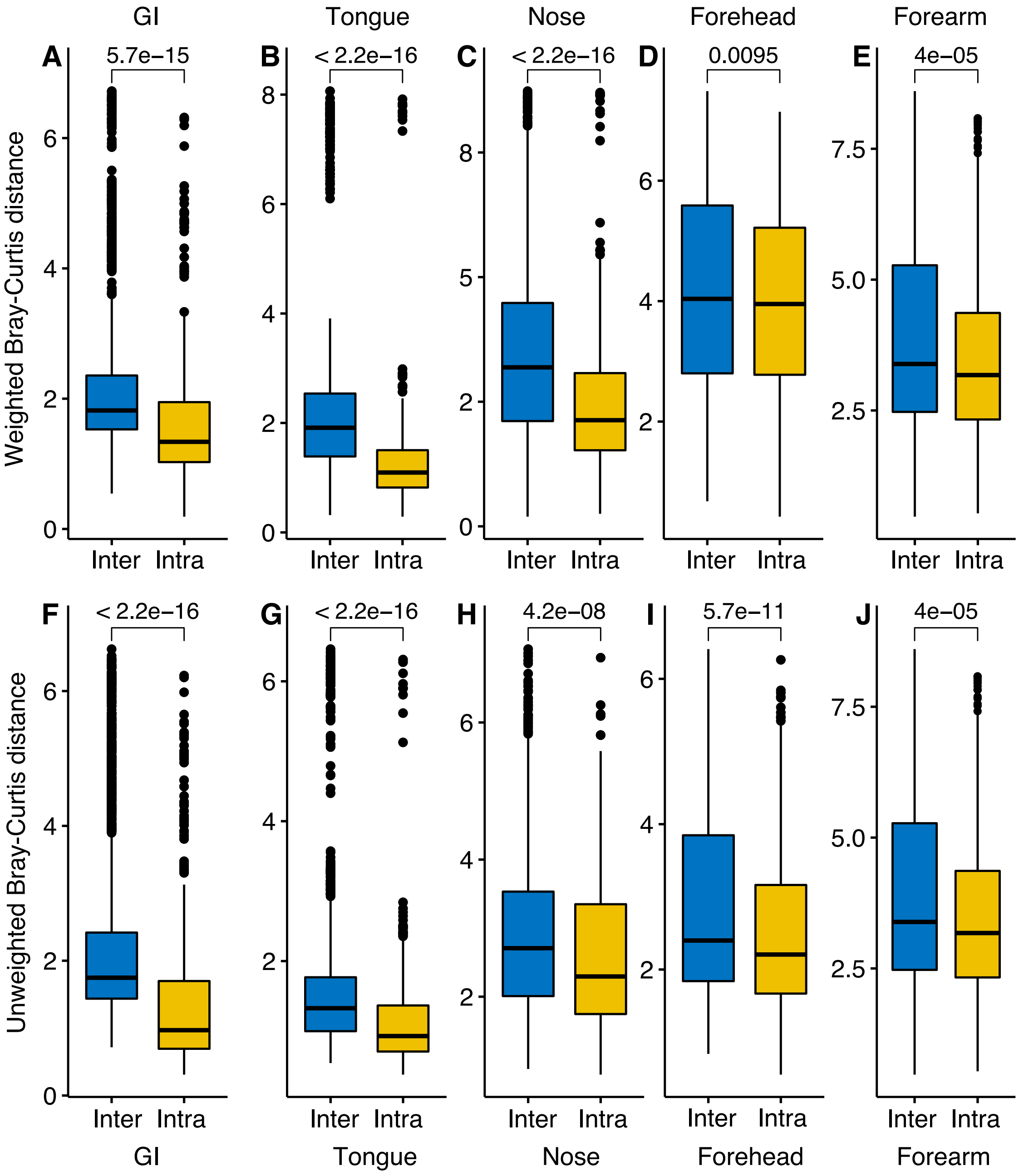

GI
Tongue
Nose
Forehead
Forearm
A
5.7e−15
B
< 2.2e−16
C
< 2.2e−16
D
0.0095
E
4e−05
Weighted Bray-Curtis distance
Inter
Intra
F
< 2.2e−16
G
< 2.2e−16
H
4.2e−08
I
5.7e−11
J
4e−05
Unweighted Bray-Curtis distance
GI
Tongue
Nose
Forehead
Forearm

Figure S3

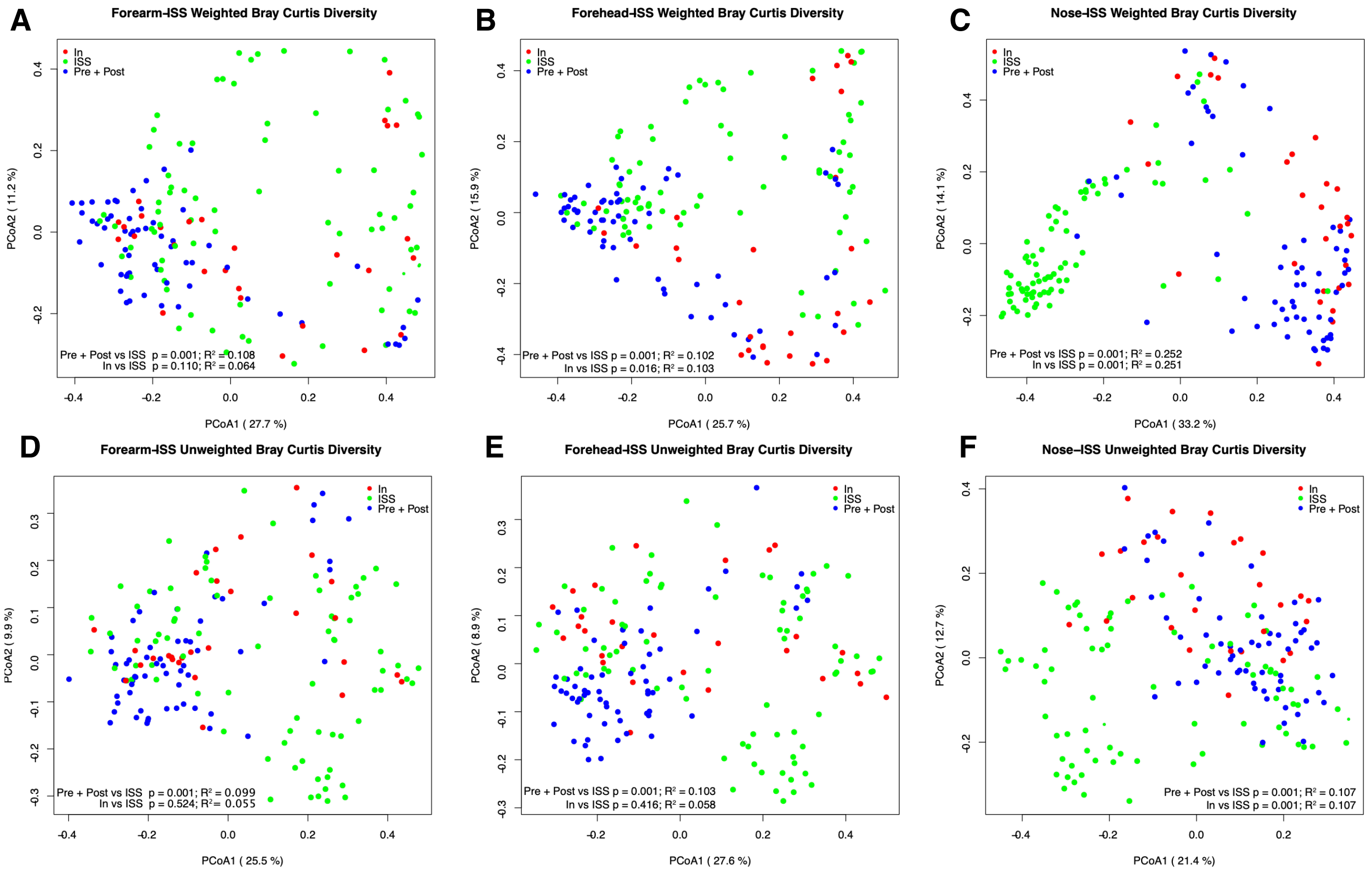
A
Forearm-ISS Weighted Bray Curtis Diversity
In
ISS
Pre + Post
Pre + Post vs ISS p = 0.001; R² = 0.108
In vs ISS p = 0.110; R² = 0.064
PCoA1 (27.7 %)
PCoA2 (11.2 %)
B
Forehead-ISS Weighted Bray Curtis Diversity
In
ISS
Pre + Post
Pre + Post vs ISS p = 0.001; R² = 0.102
In vs ISS p = 0.016; R² = 0.103
PCoA1 (25.7 %)
PCoA2 (15.9 %)
C
Nose-ISS Weighted Bray Curtis Diversity
In
ISS
Pre + Post
Pre + Post vs ISS p = 0.001; R² = 0.252
In vs ISS p = 0.001; R² = 0.251
PCoA1 (33.2 %)
PCoA2 (14.1 %)
D
Forearm-ISS Unweighted Bray Curtis Diversity
In
ISS
Pre + Post
Pre + Post vs ISS p = 0.001; R² = 0.099
In vs ISS p = 0.524; R² = 0.055
PCoA1 (25.5 %)
PCoA2 (9.9 %)
E
Forehead-ISS Unweighted Bray Curtis Diversity
In
ISS
Pre + Post
Pre + Post vs ISS p = 0.001; R² = 0.103
In vs ISS p = 0.416; R² = 0.058
PCoA1 (27.6 %)
PCoA2 (8.9 %)
F
Nose–ISS Unweighted Bray Curtis Diversity
In
ISS
Pre + Post
Pre + Post vs ISS p = 0.001; R² = 0.107
In vs ISS p = 0.001; R² = 0.107
PCoA1 (21.4 %)
PCoA2 (12.7 %)

Figure S4

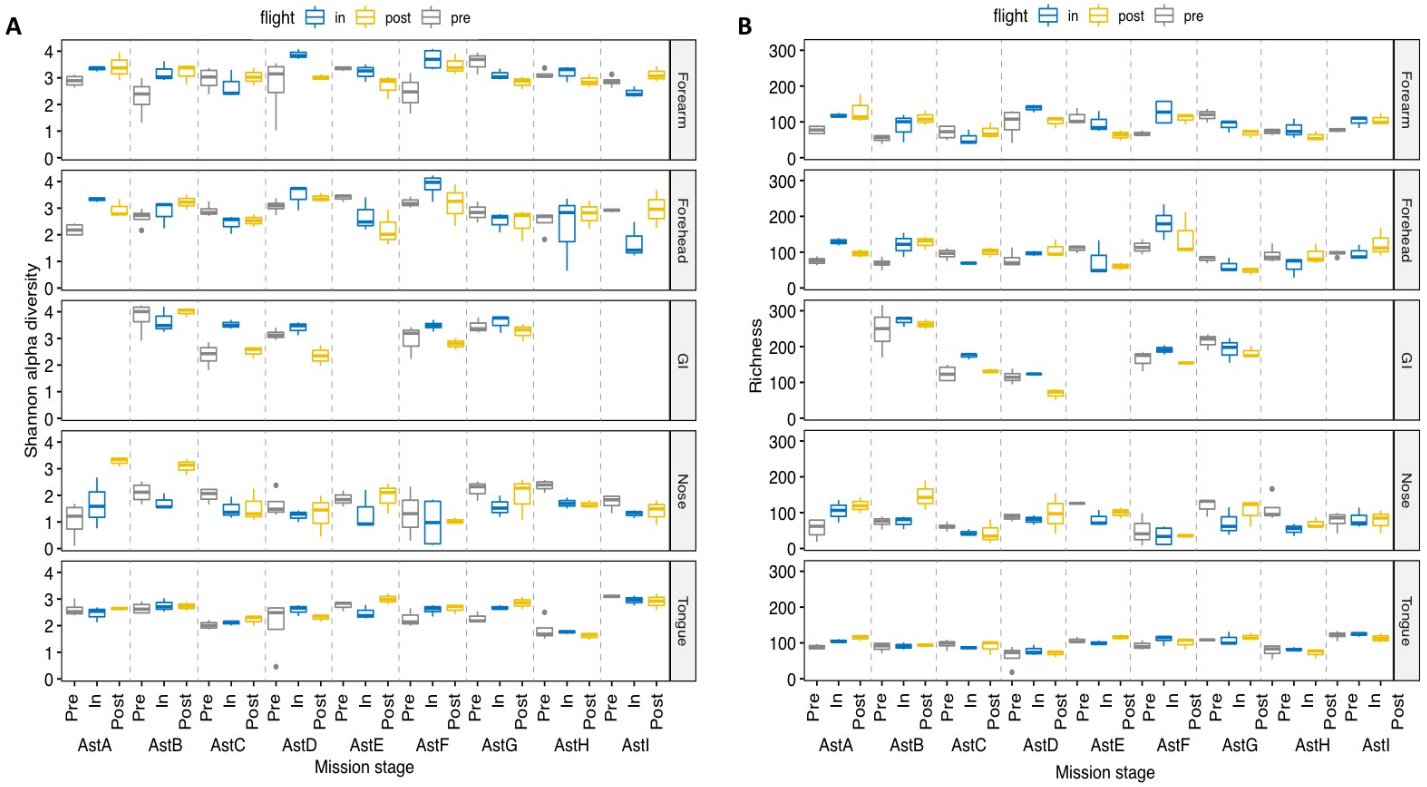
A
B
flight in post pre
Shannon alpha diversity
Richness
Forearm
Forehead
GI
Nose
Tongue
Pre In Post
AstA AstB AstC AstD AstE AstF AstG AstH AstI
Mission stage

Figure S5

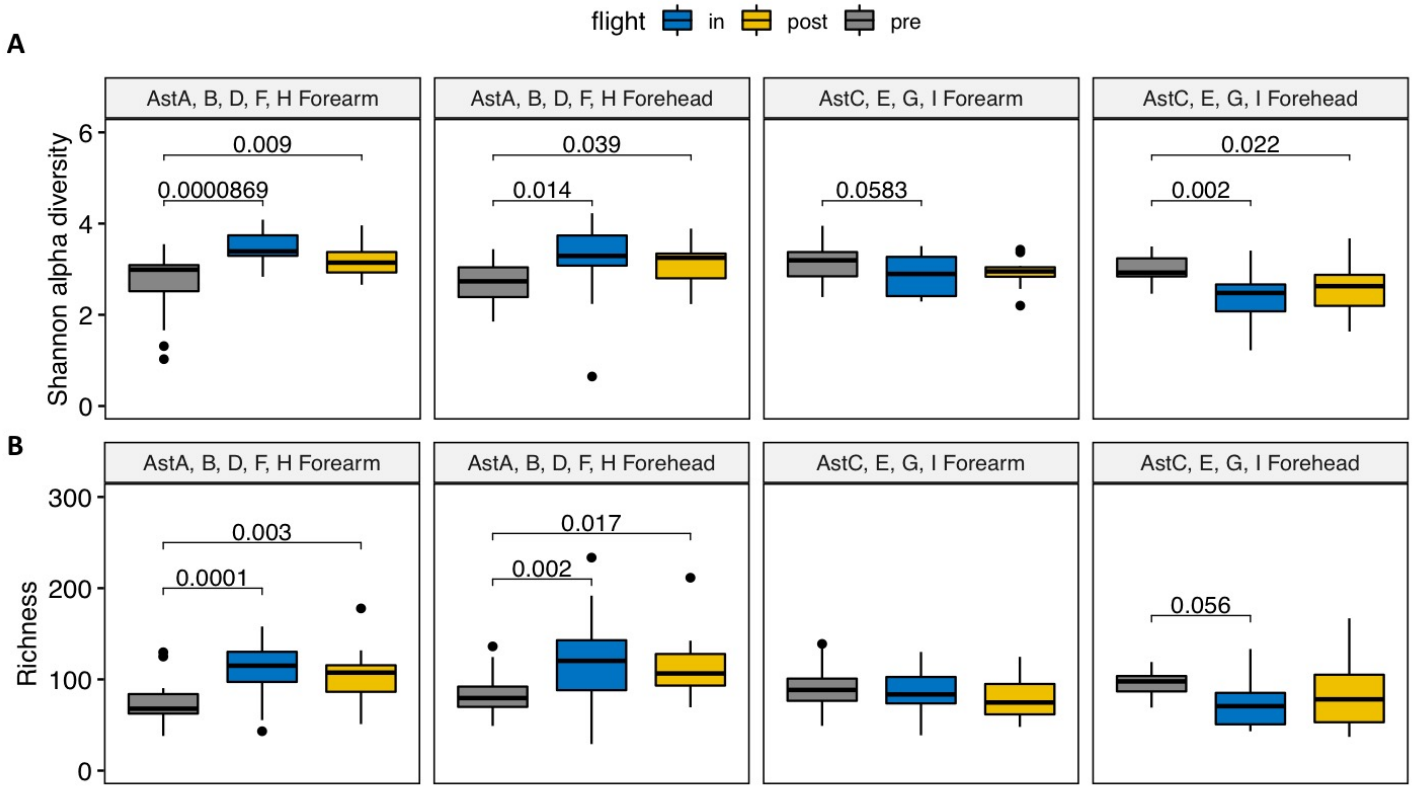
flight
in
post
pre
A
AstA, B, D, F, H Forearm
AstA, B, D, F, H Forehead
AstC, E, G, I Forearm
AstC, E, G, I Forehead
Shannon alpha diversity
0.009
0.0000869
0.039
0.014
0.0583
0.022
0.002
B
Richness
0.003
0.0001
0.017
0.002
0.056

Figure S6

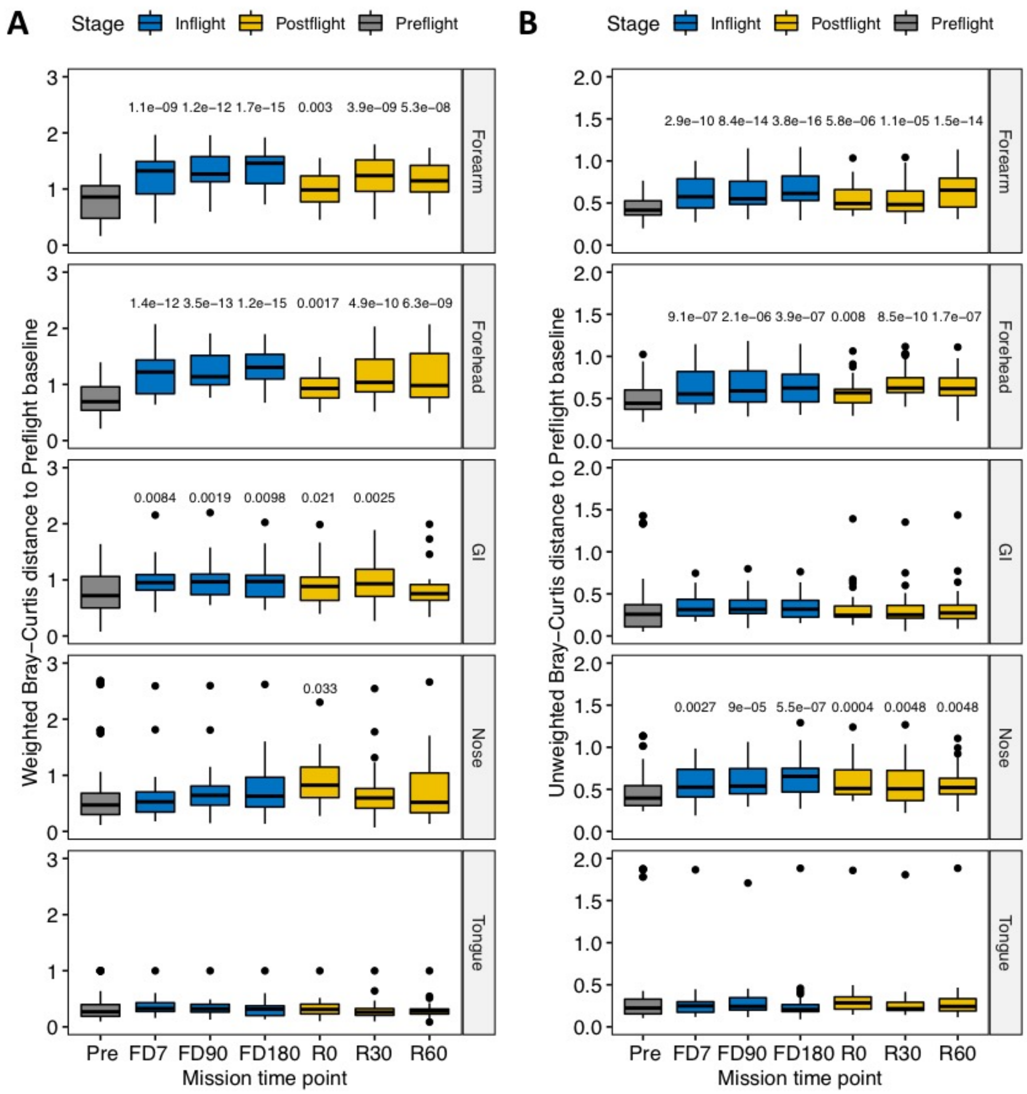
A
Stage Inflight Postflight Preflight
1.1e−09 1.2e−12 1.7e−15 0.003 3.9e−09 5.3e−08
1.4e−12 3.5e−13 1.2e−15 0.0017 4.9e−10 6.3e−09
0.0084 0.0019 0.0098 0.021 0.0025
0.033
Forearm
Forehead
GI
Nose
Tongue
Weighted Bray−Curtis distance to Preflight baseline
Pre FD7 FD90 FD180 R0 R30 R60
Mission time point
B
Stage Inflight Postflight Preflight
2.9e−10 8.4e−14 3.8e−16 5.8e−06 1.1e−05 1.5e−14
9.1e−07 2.1e−06 3.9e−07 0.008 8.5e−10 1.7e−07
0.0027 9e−05 5.5e−07 0.0004 0.0048 0.0048
Unweighted Bray−Curtis distance to Preflight baseline
Pre FD7 FD90 FD180 R0 R30 R60
Mission time point

Figure S7

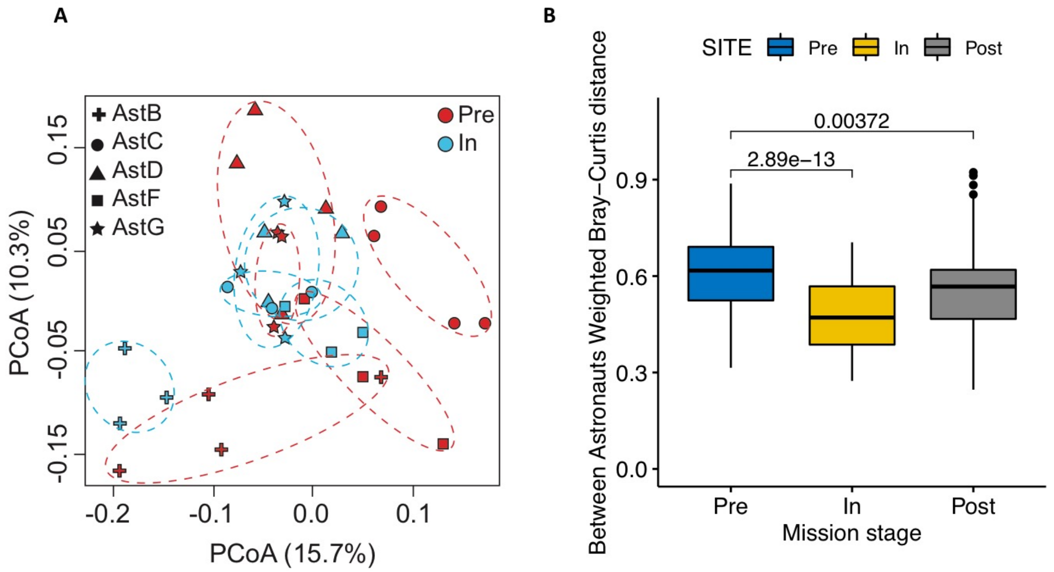
A
AstB
AstC
AstD
AstF
AstG
Pre
In
PCoA (10.3%)
PCoA (15.7%)
0.15
0.05
-0.05
-0.15
-0.2
-0.1
0.0
0.1
B
SITE
Pre
In
Post
0.00372
2.89e−13
Between Astronauts Weighted Bray−Curtis distance
0.9
0.6
0.3
0.0
Pre
In
Post
Mission stage

Figure S8

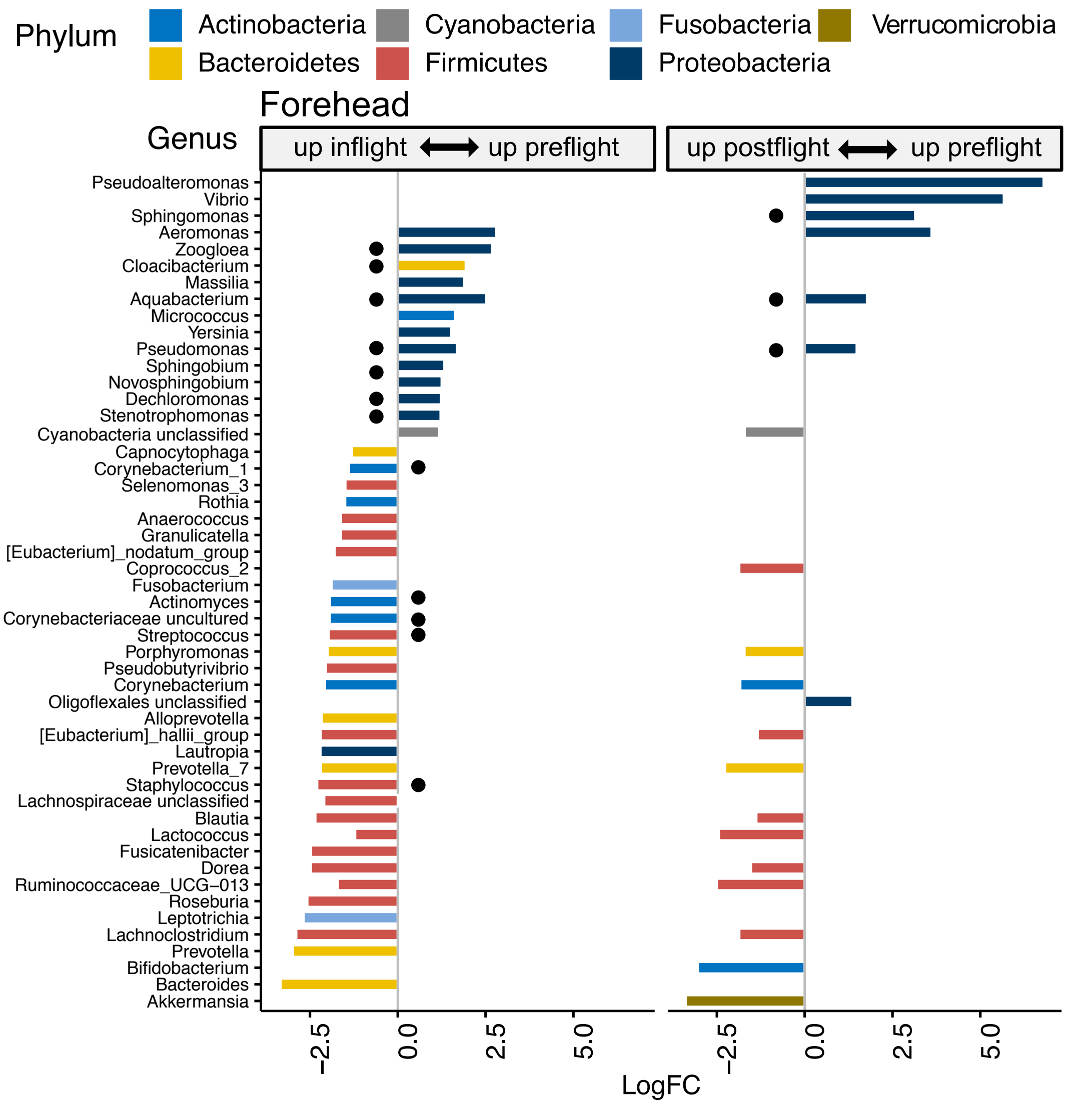

Phylum
Actinobacteria
Cyanobacteria
Fusobacteria
Verrucomicrobia
Bacteroidetes
Firmicutes
Proteobacteria
Forehead
Genus
up inflight ⟷ up preflight
up postflight ⟷ up preflight
Pseudoalteromonas
Vibrio
Sphingomonas
Aeromonas
Zoogloea
Cloacibacterium
Massilia
Aquabacterium
Micrococcus
Yersinia
Pseudomonas
Sphingobium
Novosphingobium
Dechloromonas
Stenotrophomonas
Cyanobacteria unclassified
Capnocytophaga
Corynebacterium_1
Selenomonas_3
Rothia
Anaerococcus
Granulicatella
[Eubacterium]_nodatum_group
Coprococcus_2
Fusobacterium
Actinomyces
Corynebacteriaceae uncultured
Streptococcus
Porphyromonas
Pseudobutyrivibrio
Corynebacterium
Oligoflexales unclassified
Alloprevotella
[Eubacterium]_hallii_group
Lautropia
Prevotella_7
Staphylococcus
Lachnospiraceae unclassified
Blautia
Lactococcus
Fusicatenibacter
Dorea
Ruminococcaceae_UCG−013
Roseburia
Leptotrichia
Lachnoclostridium
Prevotella
Bifidobacterium
Bacteroides
Akkermansia
−2.5
0.0
2.5
5.0
LogFC

Figure S9

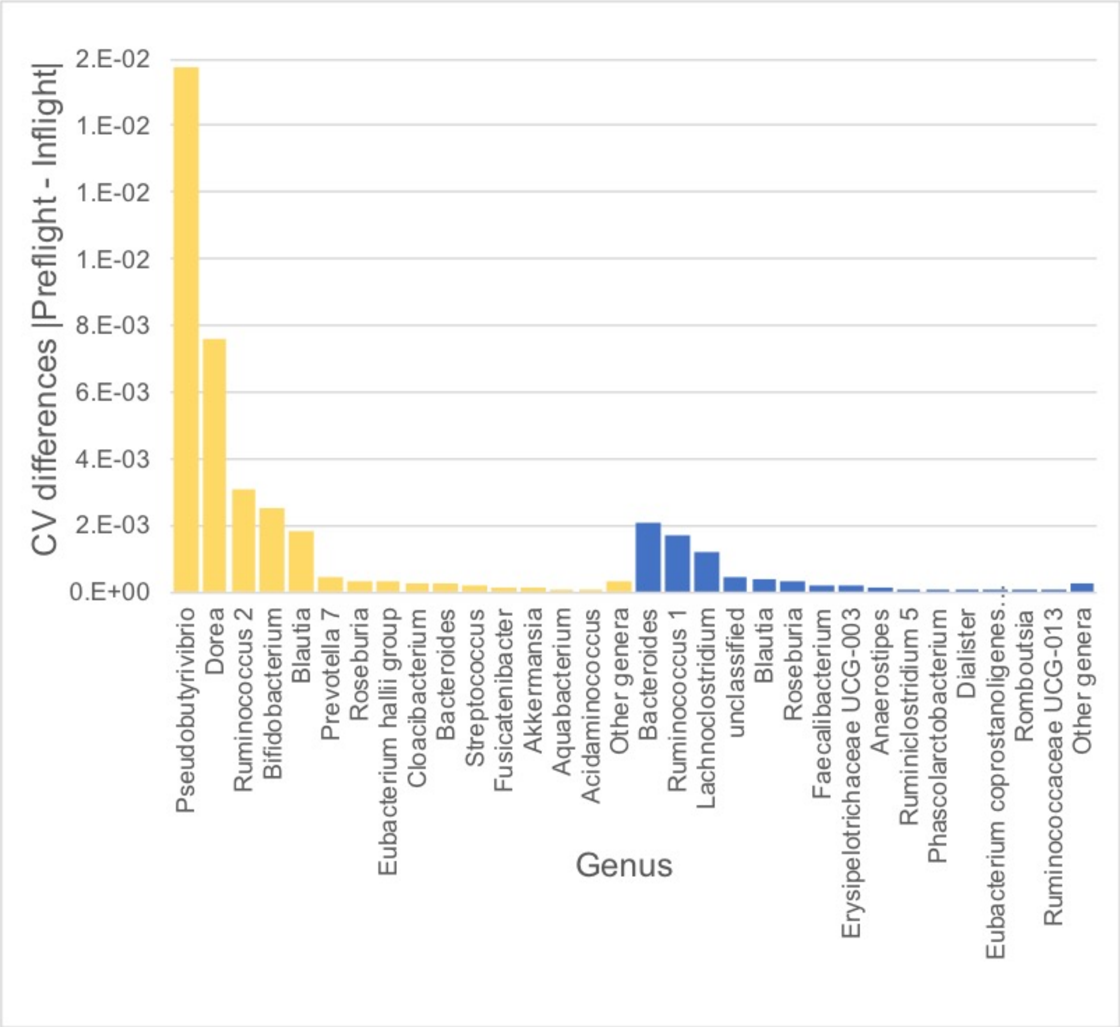

CV differences |Preflight - Inflight|
2.E-02
1.E-02
1.E-02
1.E-02
8.E-03
6.E-03
4.E-03
2.E-03
0.E+00
Pseudobutyrivibrio
Dorea
Ruminococcus 2
Bifidobacterium
Blautia
Prevotella 7
Roseburia
Eubacterium hallii group
Cloacibacterium
Bacteroides
Streptococcus
Fusicatenibacter
Akkermansia
Aquabacterium
Acidaminococcus
Other genera
Bacteroides
Ruminococcus 1
Lachnoclostridium
unclassified
Blautia
Roseburia
Faecalibacterium
Erysipelotrichaceae UCG-003
Anaerostipes
Ruminiclostridium 5
Phascolarctobacterium
Dialister
Eubacterium coprostanoligenes...
Romboutsia
Ruminococcaceae UCG-013
Other genera
Genus

Figure S10

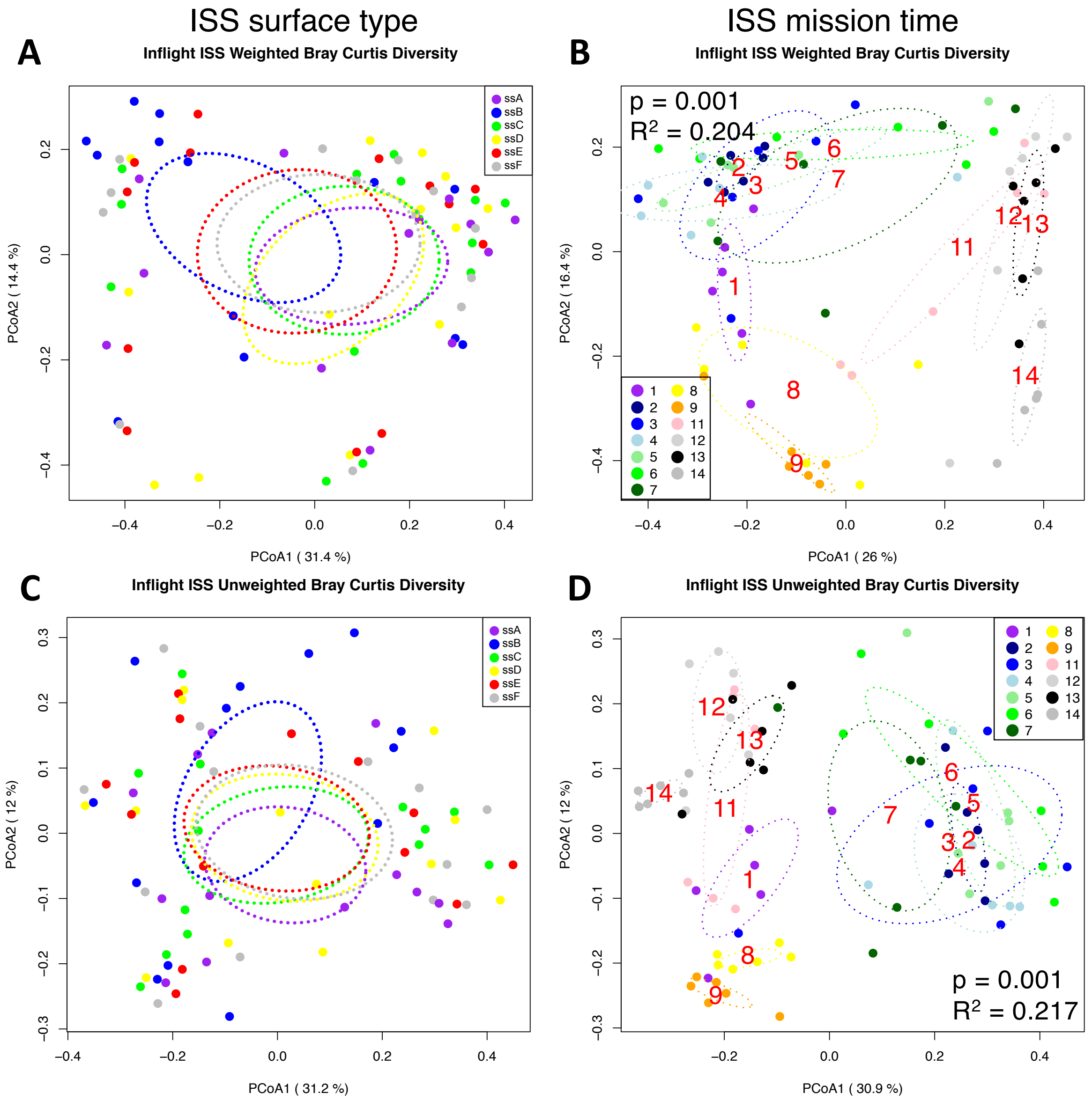

ISS surface type
A
Inflight ISS Weighted Bray Curtis Diversity
ssA
ssB
ssC
ssD
ssE
ssF
PCoA1 (31.4 %)
PCoA2 (14.4 %)
ISS mission time
B
Inflight ISS Weighted Bray Curtis Diversity
p = 0.001
R² = 0.204
PCoA1 (26 %)
PCoA2 (16.4 %)
C
Inflight ISS Unweighted Bray Curtis Diversity
PCoA1 (31.2 %)
PCoA2 (12 %)
D
Inflight ISS Unweighted Bray Curtis Diversity
p = 0.001
R² = 0.217
PCoA1 (30.9 %)
PCoA2 (12 %)

Figure S11

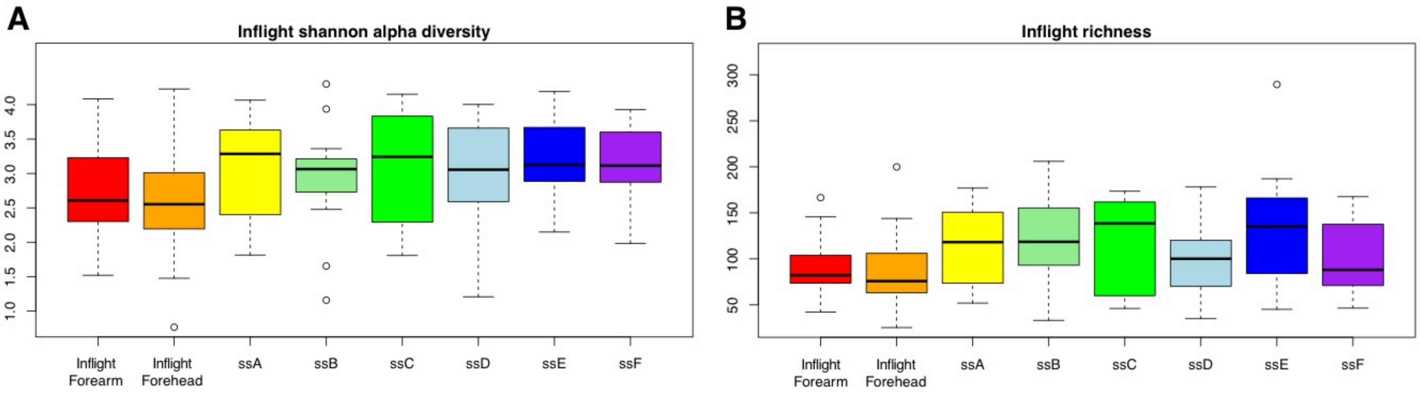

ISS surfaces sampled:

ssA: ARED Handle Bar
ssB: Intermodular Ventilation (IMV) Inlet
ssC: Cupola Nadir Window Shade Knob
ssD: Crew Quarters Stationary Light Knob
ssE: Smoke Detector
ssF: Handheld Microphone handle/grip

Figure S12

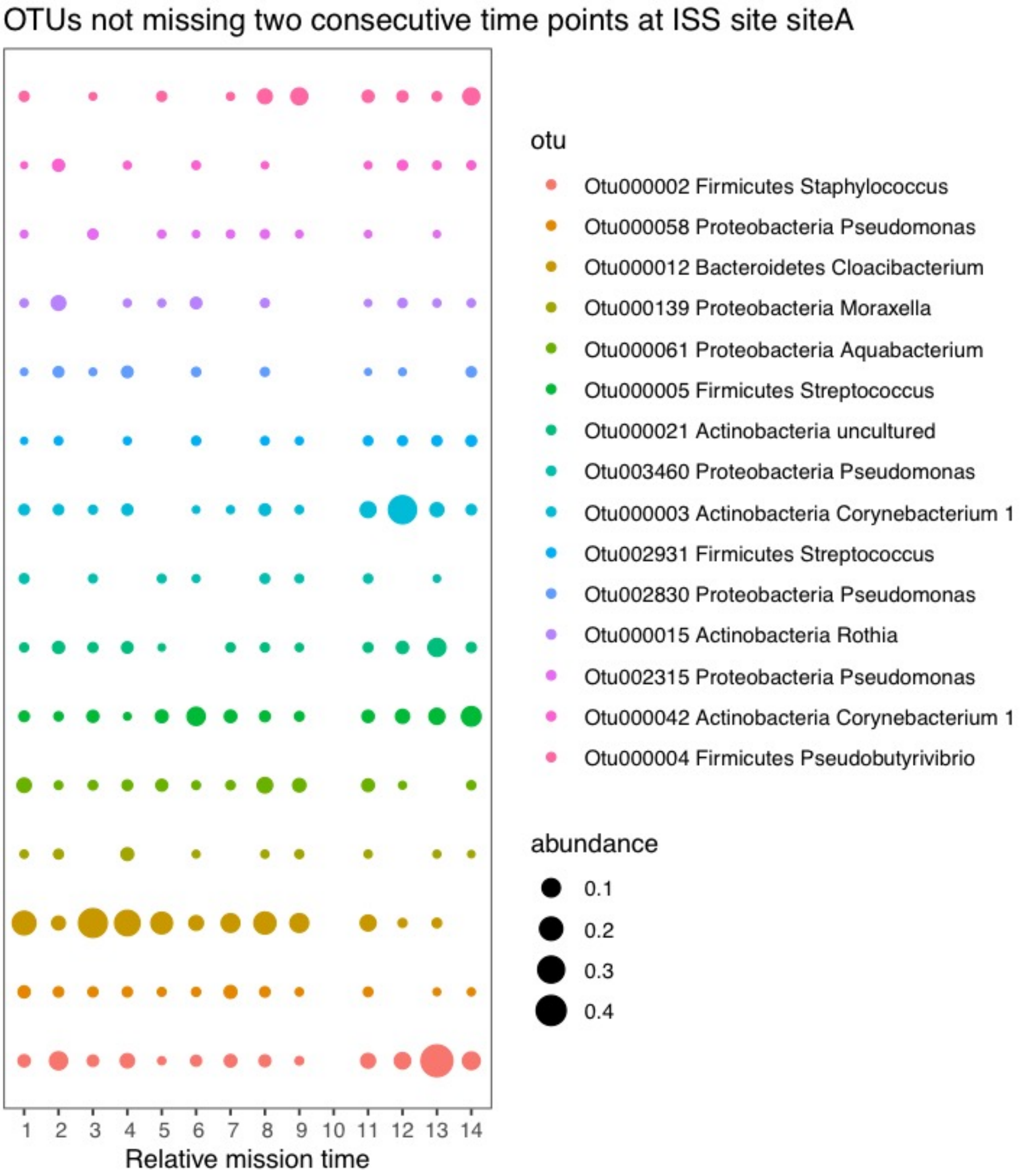
OTUs not missing two consecutive time points at ISS site siteA
otu
Otu000002 Firmicutes Staphylococcus
Otu000058 Proteobacteria Pseudomonas
Otu000012 Bacteroidetes Cloacibacterium
Otu000139 Proteobacteria Moraxella
Otu000061 Proteobacteria Aquabacterium
Otu000005 Firmicutes Streptococcus
Otu000021 Actinobacteria uncultured
Otu003460 Proteobacteria Pseudomonas
Otu000003 Actinobacteria Corynebacterium 1
Otu002931 Firmicutes Streptococcus
Otu002830 Proteobacteria Pseudomonas
Otu000015 Actinobacteria Rothia
Otu002315 Proteobacteria Pseudomonas
Otu000042 Actinobacteria Corynebacterium 1
Otu000004 Firmicutes Pseudobutyrivibrio
abundance
0.1
0.2
0.3
0.4
1 2 3 4 5 6 7 8 9 10 11 12 13 14
Relative mission time

Figure S13

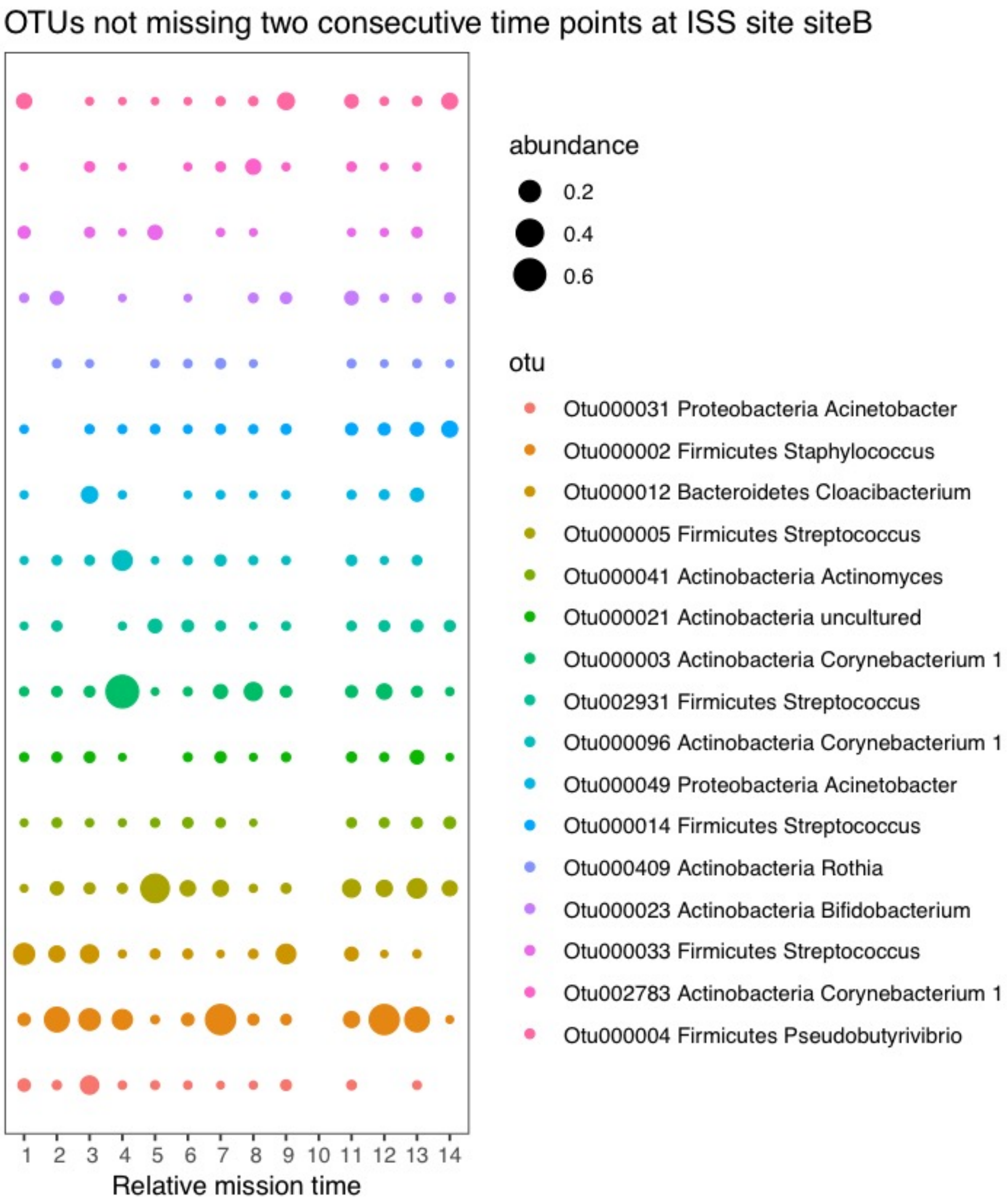

OTUs not missing two consecutive time points at ISS site siteB
abundance
0.2
0.4
0.6
otu
Otu000031 Proteobacteria Acinetobacter
Otu000002 Firmicutes Staphylococcus
Otu000012 Bacteroidetes Cloacibacterium
Otu000005 Firmicutes Streptococcus
Otu000041 Actinobacteria Actinomyces
Otu000021 Actinobacteria uncultured
Otu000003 Actinobacteria Corynebacterium 1
Otu002931 Firmicutes Streptococcus
Otu000096 Actinobacteria Corynebacterium 1
Otu000049 Proteobacteria Acinetobacter
Otu000014 Firmicutes Streptococcus
Otu000409 Actinobacteria Rothia
Otu000023 Actinobacteria Bifidobacterium
Otu000033 Firmicutes Streptococcus
Otu002783 Actinobacteria Corynebacterium 1
Otu000004 Firmicutes Pseudobutyrivibrio
1 2 3 4 5 6 7 8 9 10 11 12 13 14
Relative mission time

Figure S14

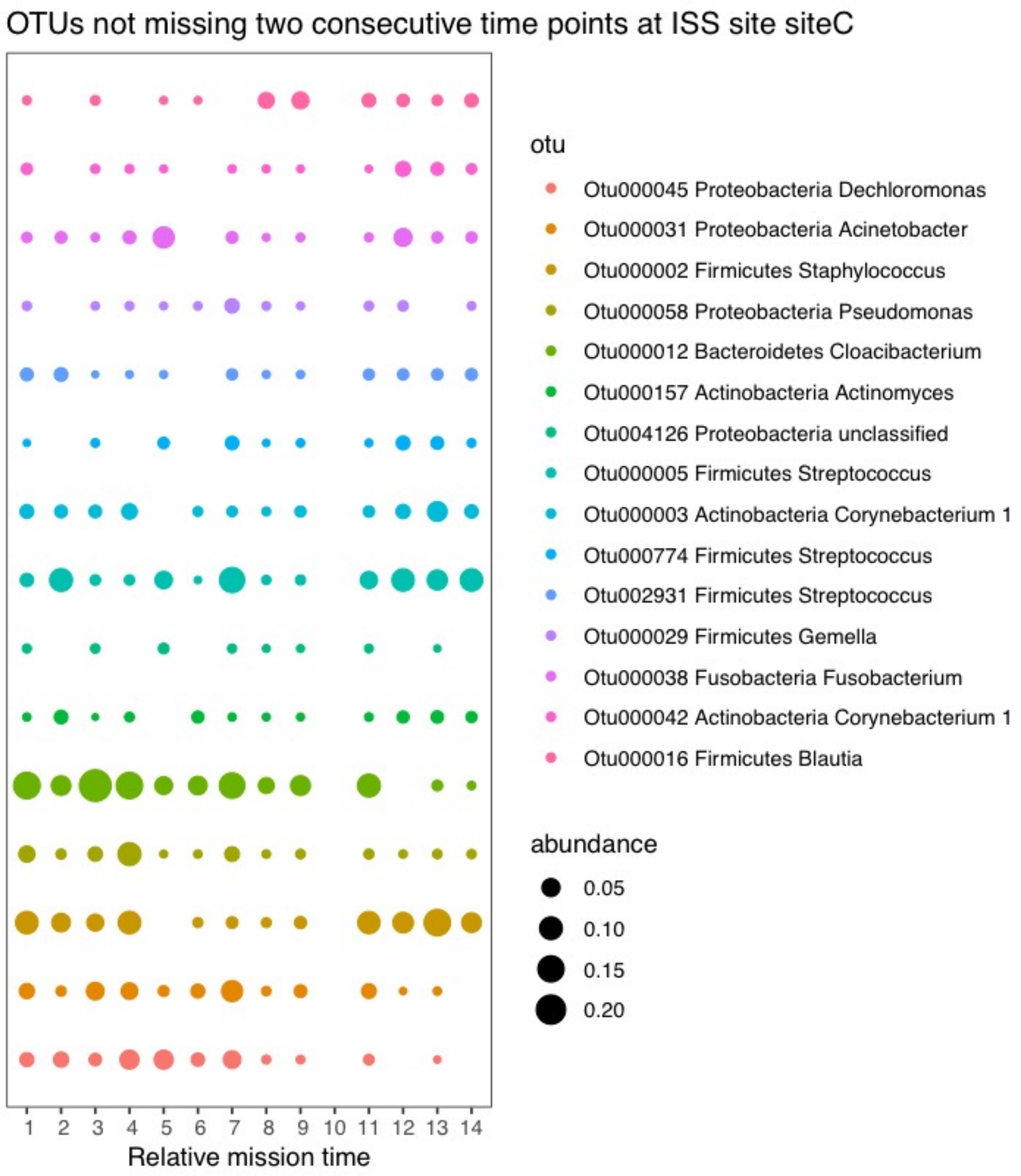

OTUs not missing two consecutive time points at ISS site siteC
otu
Otu000045 Proteobacteria Dechloromonas
Otu000031 Proteobacteria Acinetobacter
Otu000002 Firmicutes Staphylococcus
Otu000058 Proteobacteria Pseudomonas
Otu000012 Bacteroidetes Cloacibacterium
Otu000157 Actinobacteria Actinomyces
Otu004126 Proteobacteria unclassified
Otu000005 Firmicutes Streptococcus
Otu000003 Actinobacteria Corynebacterium 1
Otu000774 Firmicutes Streptococcus
Otu002931 Firmicutes Streptococcus
Otu000029 Firmicutes Gemella
Otu000038 Fusobacteria Fusobacterium
Otu000042 Actinobacteria Corynebacterium 1
Otu000016 Firmicutes Blautia
abundance
0.05
0.10
0.15
0.20
1 2 3 4 5 6 7 8 9 10 11 12 13 14
Relative mission time

Figure S15

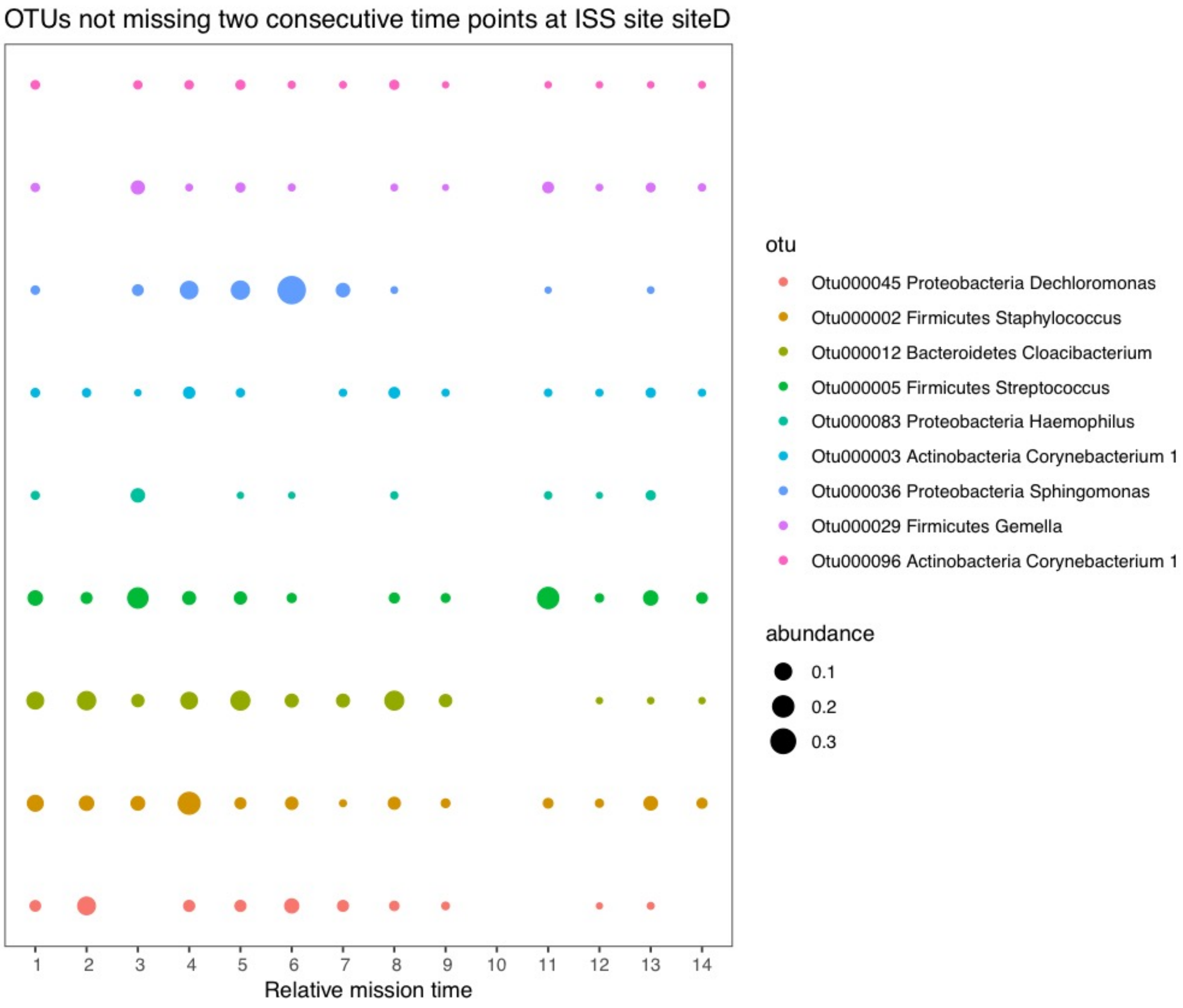
OTUs not missing two consecutive time points at ISS site siteD
1
2
3
4
5
6
7
8
9
10
11
12
13
14
Relative mission time
otu
Otu000045 Proteobacteria Dechloromonas
Otu000002 Firmicutes Staphylococcus
Otu000012 Bacteroidetes Cloacibacterium
Otu000005 Firmicutes Streptococcus
Otu000083 Proteobacteria Haemophilus
Otu000003 Actinobacteria Corynebacterium 1
Otu000036 Proteobacteria Sphingomonas
Otu000029 Firmicutes Gemella
Otu000096 Actinobacteria Corynebacterium 1
abundance
0.1
0.2
0.3

Figure S16

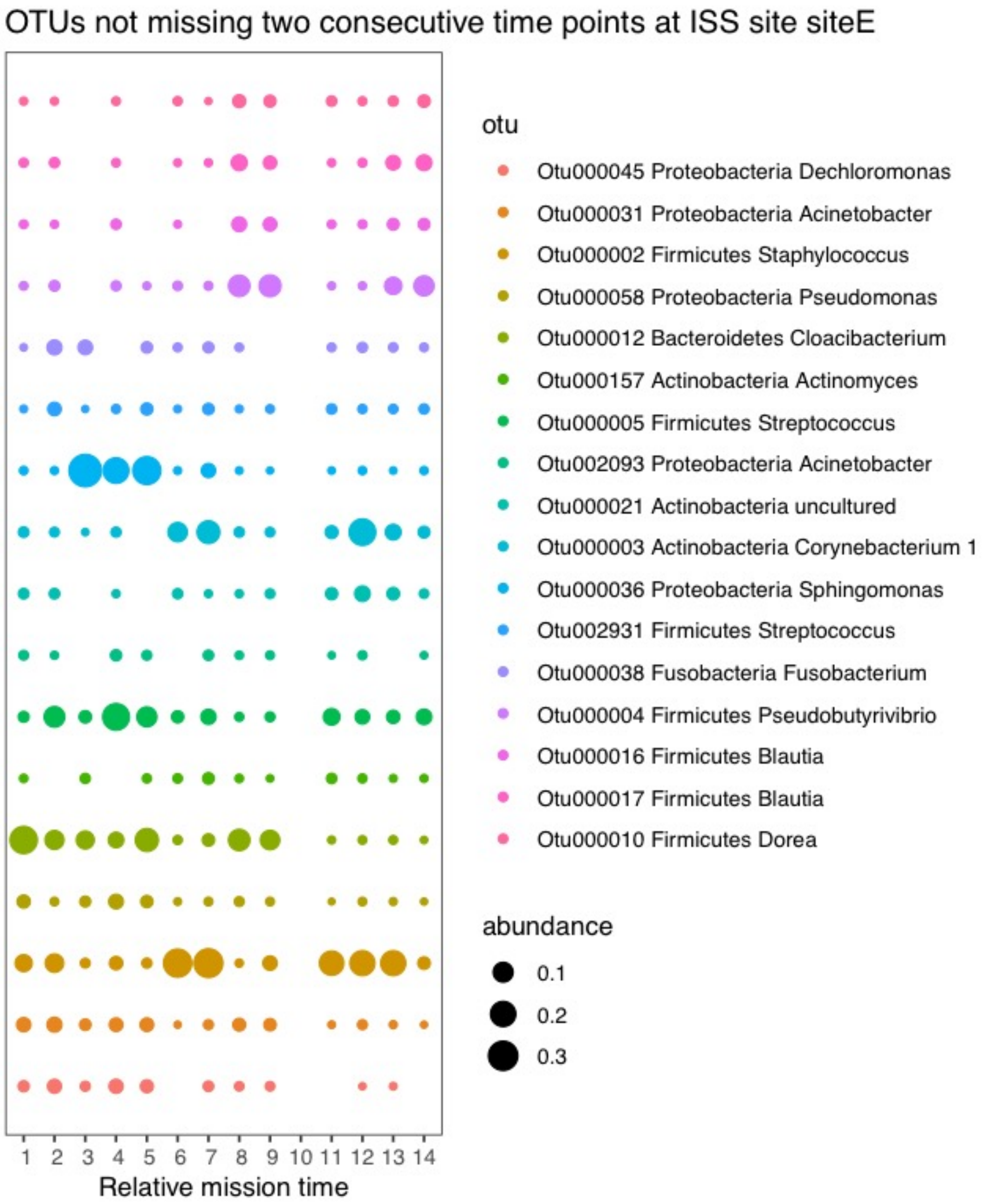

OTUs not missing two consecutive time points at ISS site siteE
otu
Otu000045 Proteobacteria Dechloromonas
Otu000031 Proteobacteria Acinetobacter
Otu000002 Firmicutes Staphylococcus
Otu000058 Proteobacteria Pseudomonas
Otu000012 Bacteroidetes Cloacibacterium
Otu000157 Actinobacteria Actinomyces
Otu000005 Firmicutes Streptococcus
Otu002093 Proteobacteria Acinetobacter
Otu000021 Actinobacteria uncultured
Otu000003 Actinobacteria Corynebacterium 1
Otu000036 Proteobacteria Sphingomonas
Otu002931 Firmicutes Streptococcus
Otu000038 Fusobacteria Fusobacterium
Otu000004 Firmicutes Pseudobutyrivibrio
Otu000016 Firmicutes Blautia
Otu000017 Firmicutes Blautia
Otu000010 Firmicutes Dorea
abundance
0.1
0.2
0.3
1 2 3 4 5 6 7 8 9 10 11 12 13 14
Relative mission time

Figure S17

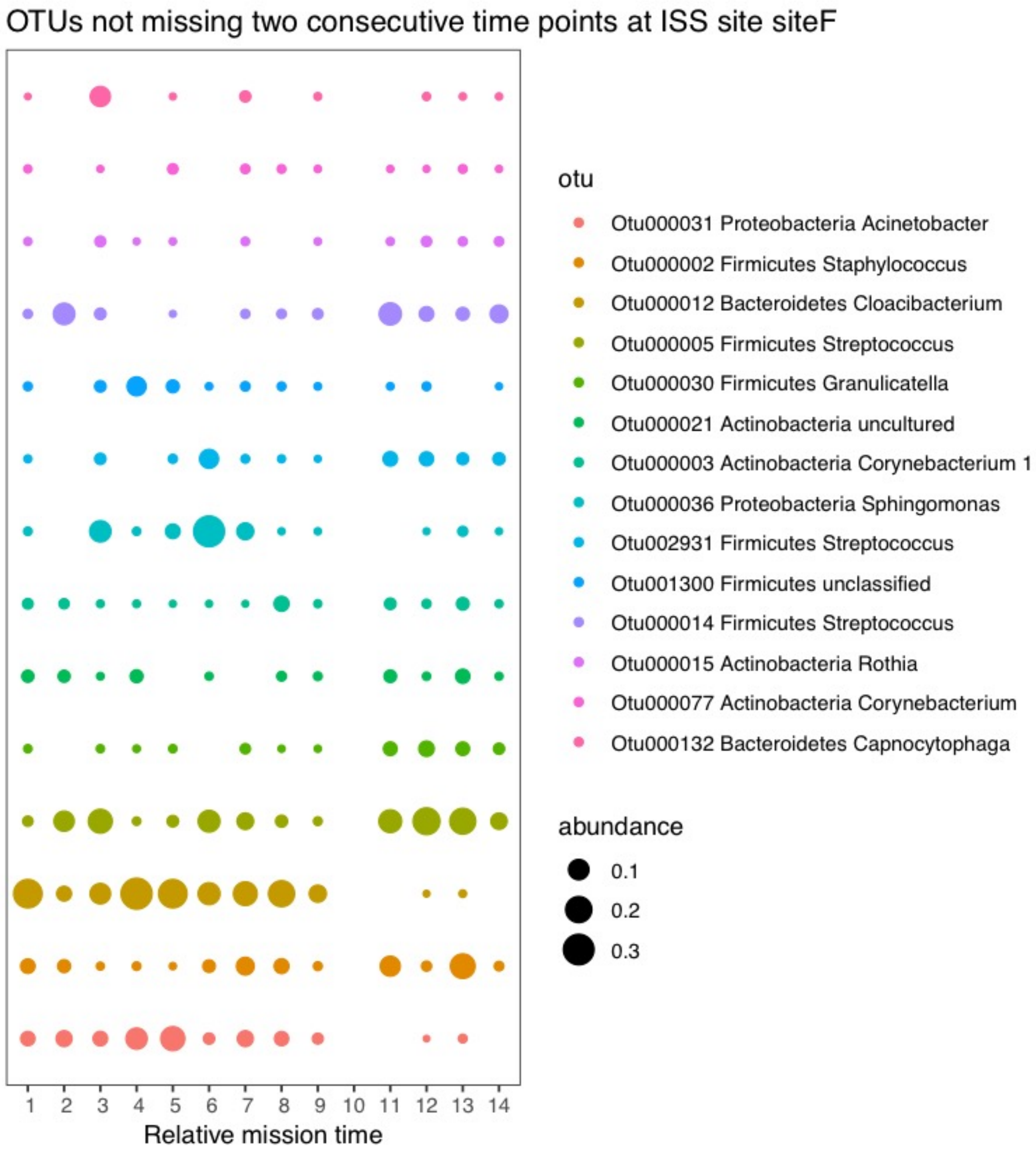
OTUs not missing two consecutive time points at ISS site siteF
otu
Otu000031 Proteobacteria Acinetobacter
Otu000002 Firmicutes Staphylococcus
Otu000012 Bacteroidetes Cloacibacterium
Otu000005 Firmicutes Streptococcus
Otu000030 Firmicutes Granulicatella
Otu000021 Actinobacteria uncultured
Otu000003 Actinobacteria Corynebacterium 1
Otu000036 Proteobacteria Sphingomonas
Otu002931 Firmicutes Streptococcus
Otu001300 Firmicutes unclassified
Otu000014 Firmicutes Streptococcus
Otu000015 Actinobacteria Rothia
Otu000077 Actinobacteria Corynebacterium
Otu000132 Bacteroidetes Capnocytophaga
abundance
0.1
0.2
0.3
1 2 3 4 5 6 7 8 9 10 11 12 13 14
Relative mission time

Figure S18

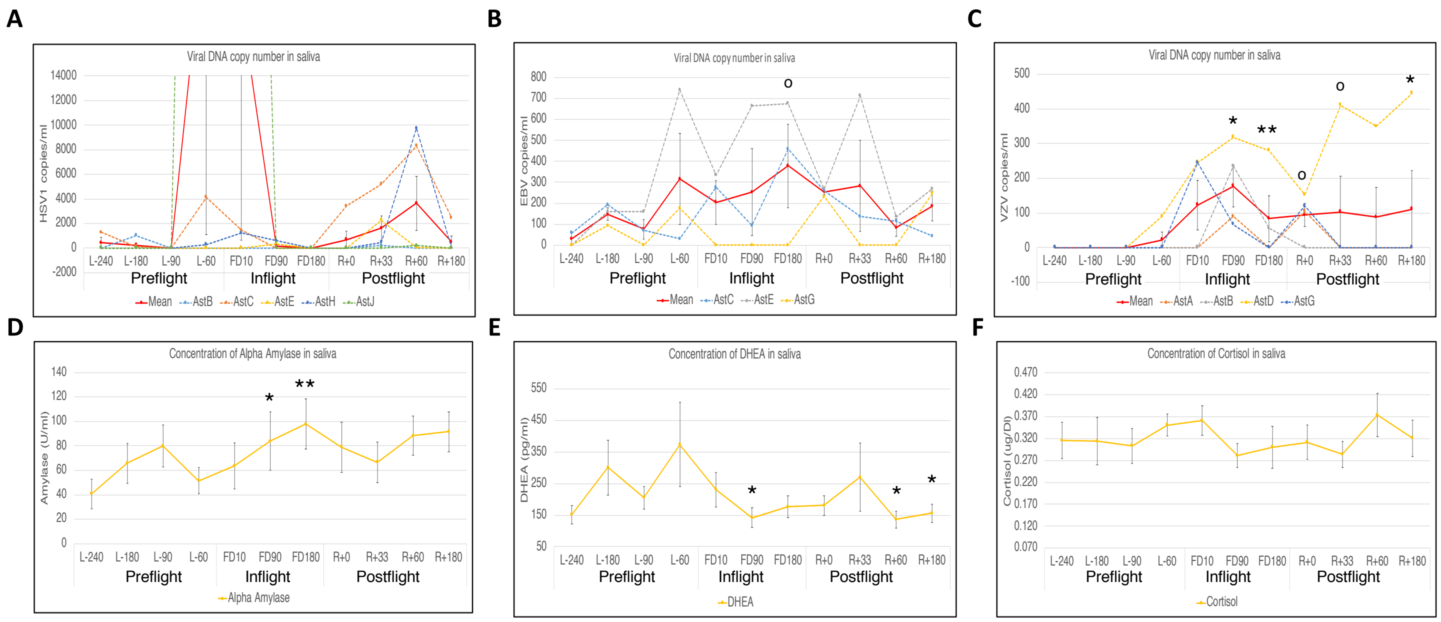

A
Viral DNA copy number in saliva
HSV1 copies/ml
14000
12000
10000
8000
6000
4000
2000
0
-2000
L-240 L-180 L-90 L-60 FD10 FD90 FD180 R+0 R+33 R+60 R+180
Preflight Inflight Postflight
Mean AstB AstC AstE AstH AstJ
B
Viral DNA copy number in saliva
EBV copies/ml
800
700
600
500
400
300
200
100
0
o
L-240 L-180 L-90 L-60 FD10 FD90 FD180 R+0 R+33 R+60 R+180
Preflight Inflight Postflight
Mean AstC AstE AstG
C
Viral DNA copy number in saliva
VZV copies/ml
500
400
300
200
100
0
-100
* ** o o *
L-240 L-180 L-90 L-60 FD10 FD90 FD180 R+0 R+33 R+60 R+180
Preflight Inflight Postflight
Mean AstA AstB AstD AstG
D
Concentration of Alpha Amylase in saliva
Amylase (U/ml)
140
120
100
80
60
40
20
0
* **
L-240 L-180 L-90 L-60 FD10 FD90 FD180 R+0 R+33 R+60 R+180
Preflight Inflight Postflight
Alpha Amylase
E
Concentration of DHEA in saliva
DHEA (pg/ml)
550
450
350
250
150
50
* * *
L-240 L-180 L-90 L-60 FD10 FD90 FD180 R+0 R+33 R+60 R+180
Preflight Inflight Postflight
DHEA
F
Concentration of Cortisol in saliva
Cortisol (ug/Dl)
0.470
0.420
0.370
0.320
0.270
0.220
0.170
0.120
0.070
L-240 L-180 L-90 L-60 FD10 FD90 FD180 R+0 R+33 R+60 R+180
Preflight Inflight Postflight
Cortisol
