# Supplementary material for: Study of the impact of long-duration space missions at the International Space Station on the astronaut microbiome
Source: Sci Rep. 2019 Jul 9;9:9911. doi: 10.1038/s41598-019-46303-8 (PMC6616552; doi:10.1038/s41598-019-46303-8)
Supplement: Supplementary file 3 — Supplementary Table S2 [file 41598_2019_46303_MOESM3_ESM.docx]

| **Cytokine** | **Transformation** | **Statistical Model*** |
| --- | --- | --- |
| **CCL2/MCP-1** | y = log(x) | MER |
| **CCL3/MIP-1a** | y = log(x) | MER |
| **CCL4/MIP-1B** | y = 1/x | MER |
| **CCL5/RANTES** | y = log(21000-x) | MER |
| **CXCL5/ENA-78** | y = log(x) | MER |
| **CXCL8/IL-8** | y = log(x) | MER |
| **FGF basic** | y = log(x) | MER |
| **G-CSF** | y=1/x | CLN |
| **GM-CSF** | y = log(x) | MER |
| **IFNg** | y=1/x | CLN |
| **IL-10** | y=1/x | CLN |
| **IL-17** | y = log(x) | MER |
| **IL-1B** | y = 1/x | MER |
| **IL-1a** | y = log(x) | MER |
| **IL-1ra** | y = log(x) | MER |
| **IL-2** | y = log(x) | not enough data |
| **IL-4** | y = log(x) | not enough data |
| **IL-5** | y = 1/sqrt(x) | MER |
| **IL-6** | y = log(x) | MER |
| **TNFa** | y = log(x) | MER |
| **Tpo** | y = log(x) | MER |
| **VEGF** | y = sqrt(x) | MER |

**Table S2:** transformation of cytokine expression values for statistical analysis.

*MER = Mixed-effects regression; CLN = Censored lognormal regression
